# The impact of anionic polymers on gene delivery: how composition and assembly help evading the toxicity-efficiency dilemma

**DOI:** 10.1186/s12951-021-00994-2

**Published:** 2021-09-27

**Authors:** Friederike Richter, Katharina Leer, Liam Martin, Prosper Mapfumo, Jana I. Solomun, Maren T. Kuchenbrod, Stephanie Hoeppener, Johannes C. Brendel, Anja Traeger

**Affiliations:** 1 Laboratory of Organic and Macromolecular Chemistry (IOMC), Friedrich Schiller University Jena, Humboldtstrasse 10, 07743 Jena, Germany; 2 Jena Center for Soft Matter (JCSM), Friedrich Schiller University Jena, Philosophenweg 7, 07743 Jena, Germany

**Keywords:** Gene delivery, Cationic polymer, Micelle, Transfection, Anionic polymer, Shielding

## Abstract

Cationic polymers have been widely studied for non-viral gene delivery due to their ability to bind genetic material and to interact with cellular membranes. However, their charged nature carries the risk of increased cytotoxicity and interaction with serum proteins, limiting their potential in vivo application. Therefore, hydrophilic or anionic shielding polymers are applied to counteract these effects. Herein, a series of micelle-forming and micelle-shielding polymers were synthesized via RAFT polymerization. The copolymer poly[(*n*-butyl acrylate)-*b*-(2-(dimethyl amino)ethyl acrylamide)] (P(*n*BA-*b*-DMAEAm)) was assembled into cationic micelles and different shielding polymers were applied, i.e., poly(acrylic acid) (PAA), poly(4-acryloyl morpholine) (PNAM) or P(NAM-*b*-AA) block copolymer. These systems were compared to a triblock terpolymer micelle comprising PAA as the middle block. The assemblies were investigated regarding their morphology, interaction with pDNA, cytotoxicity, transfection efficiency, polyplex uptake and endosomal escape. The naked cationic micelle exhibited superior transfection efficiency, but increased cytotoxicity. The addition of shielding polymers led to reduced toxicity. In particular, the triblock terpolymer micelle convinced with high cell viability and no significant loss in efficiency. The highest shielding effect was achieved by layering micelles with P(NAM-*b*-AA) supporting the colloidal stability at neutral zeta potential and completely restoring cell viability while maintaining moderate transfection efficiencies. The high potential of this micelle-layer-combination for gene delivery was illustrated for the first time.

**Supplementary Information:**

The online version contains supplementary material available at 10.1186/s12951-021-00994-2.

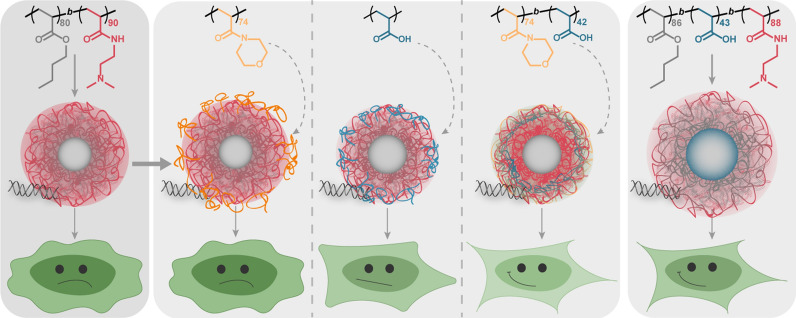

## Introduction

In the last decades, the development of non-viral nanocarriers for the (targeted) delivery of genetic material has seen great progress [1, 2], not least because of the urgent need for effective vaccines [3–5]. Optimal gene delivery vectors should be non-toxic, form stable complexes with the genetic material, exhibit low interaction with serum proteins, transfer their cargo to the desired cells, enable endosomal escape and finally ensure its activity inside the cells. Among the different non-viral gene delivery methods, cationic polymers have been investigated due to their (pH-dependent) positive charges interacting with the polyanionic genetic material and cellular membranes [6, 7]. One major advantage of using polymers is their variety due to the possibility to combine different functionalized monomers, and thus characteristics, in one polymer chain or in one assembly. Cationic polymers with vinylic backbones such as poly(meth)acrylates or poly(meth)acrylamides offer a great versatility with many monomers being commercially available, while the development of reversible deactivation radical polymerization methods (RDRP) facilitates straightforward access to various polymer architectures [8–10]. In particular, the reversible-addition fragmentation chain transfer (RAFT) polymerization is proven to be tolerant towards many functional groups under relatively simple reaction conditions, which is advantageous for the design of polymeric gene delivery vectors [11–13]. Considering nanomedicine in general, functional amphiphilic block copolymers have gained increasing attention due to their ability to self-assemble into higher ordered structures such as micelles or vesicles in aqueous solutions [14–18]. Different compositions of these polymeric micelles have been investigated for gene delivery, utilizing a cationic polymer block within their shell to form complexes with genetic material (polyplexes) [19, 20], whereas another approach uses cationic-hydrophilic block copolymers to incorporate the genetic material inside a polyion complex (PIC) with a neutral shell [14, 21]. Compared to polyplexes of cationic homopolymers, micelles have been demonstrated to increase the stability of polyplexes and the delivery efficiency [22–25], which is, among others, related to an increased interaction of the hydrophobic block with cellular membranes [26].

It is well known, that the positive charges of the cationic polymers also pose a high risk of cytotoxic side effects on cells [27–30]. This can be alleviated by designing the structure of the nanocarrier more similar to that of the plasma membrane, e.g., by the addition of hydrophilic polymers creating a steric hydration barrier around the micelle to shield the cationic charges and to decrease the interaction with serum proteins, thus prolonging circulation times within the blood [31, 32]. The most prominent example of these so-called stealth polymers is poly(ethylene glycol) (PEG), which has been incorporated into various nanocarriers for the transport of different cargos [6, 20, 31]. Nevertheless, it also has disadvantages such as the dilemma of decreasing delivery with increasing shielding efficiency [33, 34], and an accelerated blood clearance upon re-administration being linked to the occurrence of allergic reactions [35–37]. This leads to an interest in alternative stealth polymers such as poly(*N*-2-hydroxypropyl methacrylamide), poly(4-acryloylmorpholine) (PNAM) or poly(oxazoline) [31, 38, 39]. Another route towards increased cell viability is the introduction of anionic polymers, such as poly(acrylic acid) (PAA), which can reduce the positive charge density by electrostatic interaction with the cationic polymers [40]. For example, poly(methacrylic acid) (PMAA) has been incorporated into multicompartment micelles (degree of polymerization > 1000) which exhibited superior transfection efficiency while maintaining high viability [41–43]. The interaction of polyanions and polycations was also described in terms of intra- and interpolyelectrolyte complexes [44] and a layer-by-layer assembly [45]. A third possibility is the combination of both above described strategies in copolymers comprising anionic and hydrophilic blocks [46, 47], but this has not been investigated with cationic micelles so far. In general, the different shielding strategies were mostly investigated using PIC micelles or micelles/liposomes for drug delivery [48–50]. However, the opportunity of an addition of anionic polymers to cationic micelles complexed with genetic material in order to reduce toxicity while maintaining high transfection efficiency, has not been exploited so far.

Therefore, a diblock copolymer micelle with a hydrophobic core (H) of *n*-butyl acrylate (*n*BA), and a pH-responsive cationic block (C) of 2-(dimethyl amino)ethyl acrylamide) (DMAEAm) is presented in this study and used to investigate the influence of different shielding polymers on the pDNA delivery efficiency of this system. While *n*BA is well studied as a micellar core and biocompatible [51, 52], PDMAEAm has not been studied in micelles for gene delivery so far. Its low transfection efficiency as a homopolymer has certainly limited applications, but the moderate cytotoxicity make it an optimal candidate for optimization in a micellar assembly [24, 53, 54]. Different shielding polymers were added following micelle formulation (and polyplex formation) and comprised the anionic polymer poly(acrylic acid) (PAA, HC^A^), the hydrophilic stealth polymer poly(4-acryloylmorpholine) (PNAM, HC^S^), and a combination of both within a diblock copolymer P(NAM-*b*-AA) (HC^AS^). Furthermore, the performance of the layered micellar assemblies was compared to the HAC-micelle from the triblock terpolymer P(*n*BA-*b*-AA-*b*-DMAEAm), where PAA is integrated between the hydrophobic core and the cationic shell of the micelle. The physicochemical and biological properties of the assemblies were investigated using dynamic light scattering (DLS), pDNA binding and cytotoxicity assays, flow cytometry for the investigation of EGFP expression and polyplex uptake, as well as confocal laser scanning microscopy (CLSM) to investigate endosomal escape. This work aims at demonstrating the impact of the precise molecular arrangement of polymer blocks on transfection efficiency and toxicity and presents strategies for an efficient design of polymeric micelles for gene delivery.

## Main methods

Materials, instruments, detailed polymer syntheses as well as deprotection are described in the Additional file 1. Further methods such as dynamic and electrophoretic light scattering (DLS, ELS), cryo transmission electron microscopy (cryo-TEM), ethidium bromide quenching assay (EBA) and heparin dissociation assay (HRA), cytotoxicity assays, polyplex uptake and calculations are also described in the Additional file 1, since they have been performed similar as published before [23, 53].

### Synthesis and characterization

#### General procedure

All polymers investigated in this work were synthesized via RAFT polymerization. While the homopolymers PDMAEAm [53], PNAM [55] were synthesized as described previously, the synthesis of P*n*BA can be found in the Additional file 1. The block copolymers were synthesized with P*n*BA or PNAM, respectively, as macroCTAs. Briefly, the respective macroCTA, the next monomer in the block copolymer (DMAEAm or *tert-*butyl acrylate [*t*BA]), 1,4-dioxane, V-65B and 1,3,5-trioxane as an internal standard for determination of conversion by NMR were introduced to a vial equipped with a magnetic stirring bar which was sealed with a cap. The mixture was deoxygenated by bubbling argon through the solution. The vial was transferred to an oil bath set at the desired temperature. Following polymerization, the flask was cooled to room temperature (RT) and exposed to air. 2–3 droplets of the polymerization mixture were used for ^1^H NMR and SEC analysis. Afterward, the crude polymer was precipitated and dried under vacuum. Subsequently, the *t*BA containing polymers were deprotected with TFA. For exact details of each polymer, refer to the Additional file 1.

### Assembly procedure for micelle formation

#### Typical assembly procedure

A sample of P(*n*BA_80_-*b*-DMAEAm_90_) was dissolved in distilled water to reach a concentration of 75 mg mL^−1^ and treated with 0.5 equivalents of a 1 M HCl solution, followed by lyophilization. A sample of protonated P(*n*BA_80_-*b*-DMAEAm_90_) or P(*n*BA_86_-*b*-AA_43_-*b*-DMAEAm_88_) was dissolved in a mixture of THF/MeOH (80/20). Then, the same volume of a 150 mM NaCl solution in ultrapure water was added over 40 min with a syringe pump. The polymer solution was dialyzed against a 50 mM sodium acetate buffer solution (pH 5) (MWCO = 3.5–5.0 kDa). The polymer concentration was determined by lyophilization and the micelles were characterized regarding their hydrodynamic diameter and morphology by dynamic light scattering (DLS) and cryo-TEM, respectively. Experimental details for each analysis are provided in the Additional file 1. During the investigation process, different batches of micelle assemblies from the very same diblock or triblock copolymer were used for preliminary experiments. While all the results in this manuscript were performed with one batch, comparisons of the different batches can be found in the Additional file 1: Figures S6, S9, S15, Table S4)

### Preparation of (layered) polyplexes

The polyplexes were prepared similarly to literature procedures [53] in HBG buffer (20 mM 4-(2-hydroxethyl) piperazine-1-ethanesulfonic acid (HEPES) and 5% (w/v) glucose, pH 7.4), but 1.3-fold concentrated. Briefly, a 40 µg mL^−1^ solution of pDNA was mixed 1 + 1 with polymer solutions or micelle suspensions containing different quantities of polymer to vary the ratio of protonatable nitrogen atoms to phosphates of pDNA (N*/P ratio). If not stated otherwise, the pDNA concentration within the polyplex solution was 15 µg mL^−1^. Immediately after combination, the mixtures were vortexed for 10 s at maximum speed (3200 rpm) and incubated at RT for 15 min to ensure complex formation. Meanwhile, a fourfold concentrated shielding solution was prepared to give a PNAM/P*n*BA molar ratio of 1.0 or a carboxy to amine group ratio (COOH/NH-ratio) of 0.5 in the final micelle-shielding-mix. Subsequently, the polyplex solution was slowly pipetted directly into the shielding solution, giving an assembly of 75% (v/v) polyplex and 25% (v/v) layer. Where no shielding was desired, the volume was replaced by HBG buffer.

### Cell culture

The mouse fibroblast cell line L-929 and the human embryonic kidney cell line HEK293T were obtained from CLS (Germany). They were maintained as recommended by the supplier and cultured in D10 (low glucose Dulbecco’s modified eagle’s medium (DMEM) supplemented with 10% (v/v) fetal calf serum (FCS), 100 U mL^−1^ penicillin and 100 µg mL^−1^ streptomycin) at 37 °C in a humidified 5% (v/v) CO_2_ atmosphere.

### Transfection efficiency

To determine the influence of the polymers on the expression of EGFP, the HEK293T cells were seeded at 0.2 × 10^6^ cells mL^−1^ in growth medium (D10) containing 10 mM HEPES (D10H) in 24-well plates, followed by incubation at 37 °C in a humidified 5% (v/v) CO_2_ atmosphere for 24 h and a medium change to fresh D10H 1 h prior to treatment. The cells were treated with polyplexes with or without shielding at N*/P 30 and a final pDNA concentration of 1.5 µg mL^−1^ in the well. The polyplexes were prepared as described above with isolated mEGFP-N1 pDNA and added to the cells diluting the polyplexes 1:10 in the cell culture medium for an incubation period of 24 h or 48 h. For analysis via flow cytometry, HEK293T cells were harvested by transferring the supernatant to a 24-well plate, trypsinizing the cells and resuspending them in the respective supernatant again. Half of the suspension was transferred to a 96-well plate for measurement, while the remaining cell suspension was diluted 1:2 with D20 (D10 + 10% FCS) and incubated for further 24 h.

For determination of transfection efficiency, cells were analyzed as described in the instrumentation section (Additional file 1). Viable cells showing EGFP signal higher than the mock control cells incubated with polyplexes of the respective polymer and pKMyc pDNA were gated as percentage of cells expressing EGFP (Additional file 1: Figure S18) and the relative mean fluorescence intensity (rMFI) of all viable cells was calculated in relation to the respective mock control. MFI values of the controls can be found in the Additional file 1: Table S8. The experiments were performed three times and data are expressed as mean ± SD.

### Calcein release assay

To determine the endosomal escape efficiency of the polymers, a calcein release assay was performed with HEK293T cells seeded at 0.2 × 10^6^ cells mL^−1^ in D10H in 4-well glass bottom dishes. Following incubation at 37 °C in a humidified 5% (v/v) CO_2_ atmosphere for 24 h, the cells were treated with polyplexes at N*/P 30 with or without shielding. Just before the addition of polyplexes, the non-cell-permeable dye calcein was added to the cells to give a final concentration of 25 µg mL^−1^. Following incubation for 1 h, the cells were washed twice with warm FC-buffer (Hanks’ Balanced Salt Solution, supplemented with 2% FCS and 20 mM HEPES) followed by addition of fresh warm D20 and incubation with 8 µM Hoechst 33342 for 10 min. Cells were analyzed via confocal laser scanning microscopy (CLSM) and the images were processed using ImageJ, version 1.52 [56]. A detailed description can be found in the Additional file 1. The amount of cells with calcein release was quantified by determining and counting nuclei showing green calcein fluorescence in a high-throughput analysis (< 170 cells/repetition) via ImageJ:

1 $${\text{Cells with calcein release}}/{\text{\% }} = { }\frac{{\text{Number of nuclei with coincident calcein staining}}}{{\text{Number of nuclei}}}{ } \cdot {100}$$

Subsequently, the escape efficiency was calculated relative to the number of YOYO-1 positive cells at the same time point (2):

2 $${\text{Escape efficiency}}:\frac{{\text{Cells with calcein release (1 h)}}}{{\text{YOYO - 1 positive cells (1 h)}}} \cdot {100}$$

### Statistics

To determine the statistical significance, repeated measures analysis of variance (RM-ANOVA) was performed. If the RM-ANOVA revealed significant differences (*p* < 0.05), post-hoc analyses with a Bonferroni correction were applied. If not stated otherwise, statistically significant differences were indicated with * for *p* < 0.05 ** for *p* < 0.01 and with *** for *p* < 0.001. All statistical analyses were performed with data of n ≥ 3 in Origin, Version 2020b (OriginLab Corporation, US).

## Results and discussion

### Polymer synthesis and characterization

**Fig. 1:**
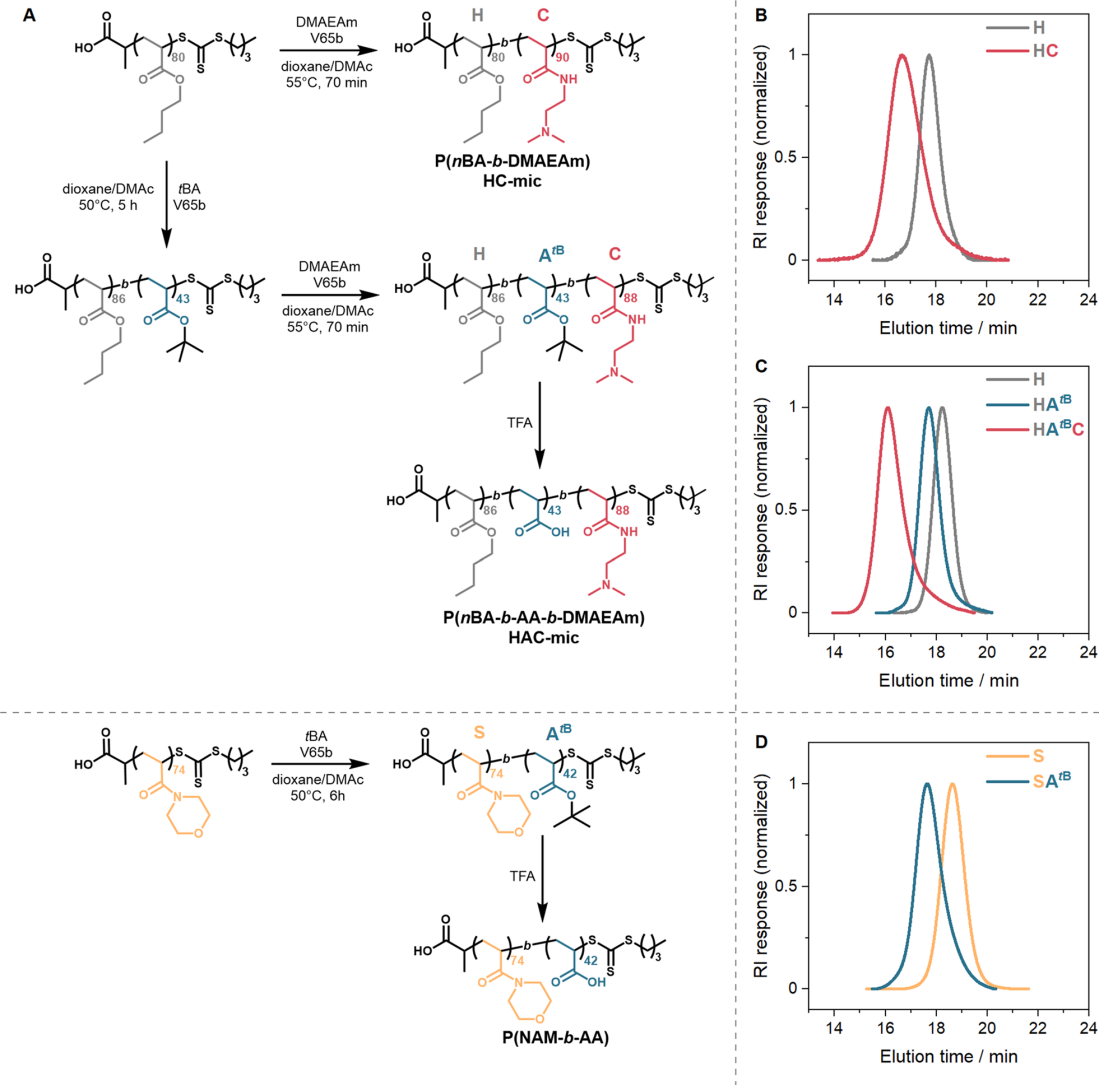
Synthesis and characterization of the block copolymers. **A** Synthesis routes of block copolymers. **B**–**D** SEC-traces of the respective polymers using a (DMAc + 0.21% LiCl)-SEC with PMMA-calibration. **B** P(*n*BA-*b*-DMAEAm) and the P*n*BA precursor, **C** P(*n*BA-*b*-*t*BA-*b*-DMAEAm) and its precursors, **D** P(NAM-*b*-*t*BA) and its precursor PNAM

The diblock and triblock copolymers were synthesized via sequential RAFT polymerizations with purification after each block synthesis step (Fig. 1A). (Propanoic acid)yl butyl trithiocarbonate (PABTC) was used as the chain transfer agent (CTA), since it is suitable to control the polymerization of acrylates and acrylamides [57]. The core-forming hydrophobic block was synthesized by polymerization of *n*-butyl acrylate (*n*BA) and was used as a macroCTA for the subsequent RAFT polymerizations. This block was chain extended (i) with the amine-functional cationic monomer dimethylaminoethyl acrylamide (DMAEAm) yielding the diblock copolymer P(*n*BA-*b*-DMAEAm), or (ii) with the carboxyl-functional anionic monomer *tert*-butyl acrylate (*t*BA), followed by chain extension with DMAEAm to give the triblock terpolymer P(*n*BA-*b*-*t*BA-*b*-DMAEAm).

A PDMAEAm homopolymer was synthesized as a control polymer as described previously [53]. Three different shielding polymers were used: (i) 2 kDa poly(acrylic acid) (PAA) which is commercially available, while (ii) poly(*N*-acryloylmorpholine) (PNAM) and (iii) a diblock copolymer comprising NAM and acrylic acid, P(NAM-*b*-AA), were synthesized by RAFT polymerization, starting with the hydrophilic PNAM followed by chain extension with *t*BA to obtain the diblock copolymer.

The size exclusion chromatography (SEC) curves in Fig. 1 display monomodal populations with relatively narrow molar mass distributions. The experimental number-average molar masses differ from the theoretical values, since the hydrodynamic radii of the applied polymers and standards utilized for calibration of the SEC systems were different (Table 1). Following characterization, P(NAM-*b*-*t*BA) and P(*n*BA-*b*-*t*BA-*b*-DMAEAm) were deprotected with TFA to expose the anionic carboxyl-group of P*t*BA, obtaining the anionic PAA block, as confirmed by ^1^H NMR spectroscopy (Additional file 1: Figures S3, S4). The molar mass distribution of P(NAM-*b*-AA) after deprotection obtained by aqueous SEC can be found in Additional file 1: Figure S4. It was not possible to measure the molar mass distribution by aqueous SEC for the diblock copolymer P(*n*BA-*b*-DMAEAm) and triblock terpolymer P(*n*BA-*b*-AA-*b*-DMAEAm) due to their amphiphilic characters.

**Table 1:** Summary of polymer characterization

Polymer-ID^a^	Assembly code	*M*_n,th_^b^ (kg mol^−1^)	*M*_n,SEC_^c^ (kg mol^−1^)	*Ð*^c^ (kg mol^−1^)
P(*n*BA_80_-*b*-DMAEAm_90_)	HC	23.3	28.4	1.37
P(*n*BA_86_-*b*-*t*BA_43_-*b*-DMAEAm_88_)	HAC	29.3	34.6	1.26
PNAM_72_	HC^S^	10.4	8.8	1.12
P(NAM_74_-*b*-*t*BA_42_)	HC^AS^	16.0	15.2	1.20
PAA	HC^A^	2.0^d^	1.0^e^	1.27^e^

^a^DP was determined via ^1^H NMR

^b^Determined using Additional file 1: Equation S1

^c^Determined via DMAc-SEC with PMMA standards

^d^According to the distributor

^e^Determined via aqueous SEC with PEG-standards

**Fig. 2:**
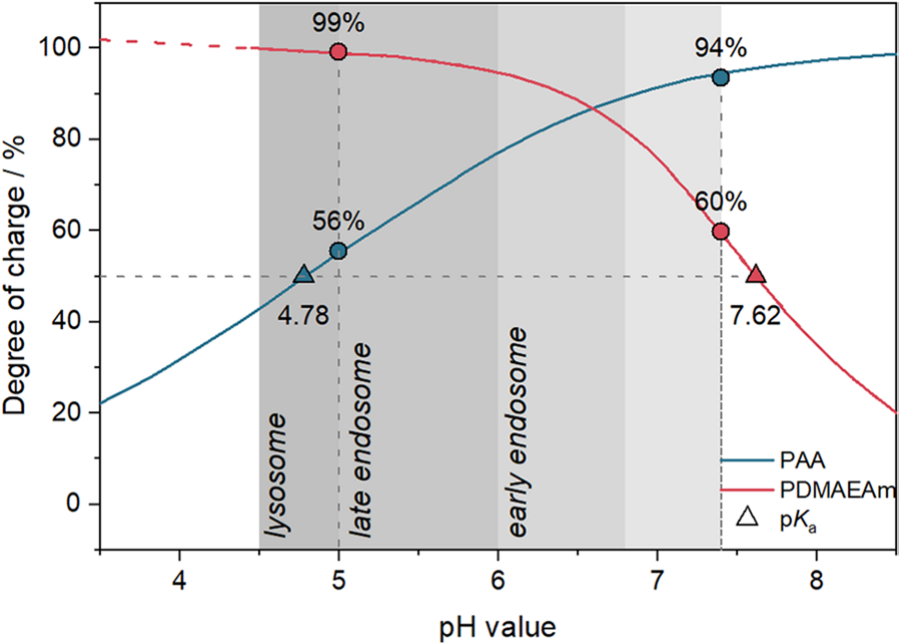
Degree of charge and p*K*_a_ values. Theoretical determination of the degree of charge based on Additional file 1: Equations S2, S3 following titration of polymers (5 mg mL^−1^ in 150 mM NaCl) against 0.1 M NaOH (PDMAEAm) or 0.1 M HCl (PAA). The red dashed line indicates a linear mathematical extrapolation of the curve. Dots represent the degree of charge at pH 5 or pH 7.4 and triangles indicate the p*K*_a_ values calculated using Additional file 1: Equation S4. The grey region designates physiologically relevant pH windows according to Mellman et al*.* [58], Huotari and Helenius [60]

To investigate the influence of different pH-values present in the biological system (e.g., pH 7.4 extracellularly and pH 5.5 in endolysosomes) [58] on the behavior and interactions of PAA and PDMAEAm, both polymers were titrated against HCl or NaOH, respectively, and the proportion of protonated amine groups (degree of charge) was calculated using Additional file 1: Equations S2, S3 (Fig. 2) [59]. At pH 7.4, PDMAEAm shows a moderate degree of charge (60%), whereas PAA is nearly completely negatively charged (94%). By contrast, the degrees of charge are reversed at pH 5.0 (PDMAEAm: 99%, PAA: 56%). These results indicate on the one hand that PAA would neutralize a majority of the remaining positive charges of PDMAEAm at pH 7.4 in the extracellular environment, which could lead to reduced cytotoxicity. On the other hand, when the pH is decreasing in the endolysosomal pathway, the reducing amount of negative charge would unleash further positive charges which is beneficial for endosomal escape.

### Micelle formation and pH dependence

Micelles of P(*n*BA-*b*-DMAEAm) and P(*n*BA-*b*-AA-*b*-DMAEAm) were prepared by gradually adding 150 mM NaCl solution as selective solvent to the polymer dissolved in the good solvent THF/MeOH (80/20 v/v). During this process, the block copolymers eventually underwent microphase separation, where P*n*BA formed the core (H—hydrophobic), and PAA (A—anionic) and/or PDMAEAm (C—cationic) the corona, resulting in the assembly of HC- and HAC-micelles, respectively. The polymer solutions were dialyzed against 50 mM sodium acetate buffer solution (pH 5.0) to replace the solvent mixture and to enhance the stability of the micelles by avoiding neutralization of the cationic charges by PAA at higher pH-values. The formation of micelles was verified first by DLS measurements (Additional file 1: Figure S6). In addition, the reproducible formulation and stability at RT for at least 1 year indicate high application potential (Additional file 1: Figures S9, S14).

**Fig. 3:**
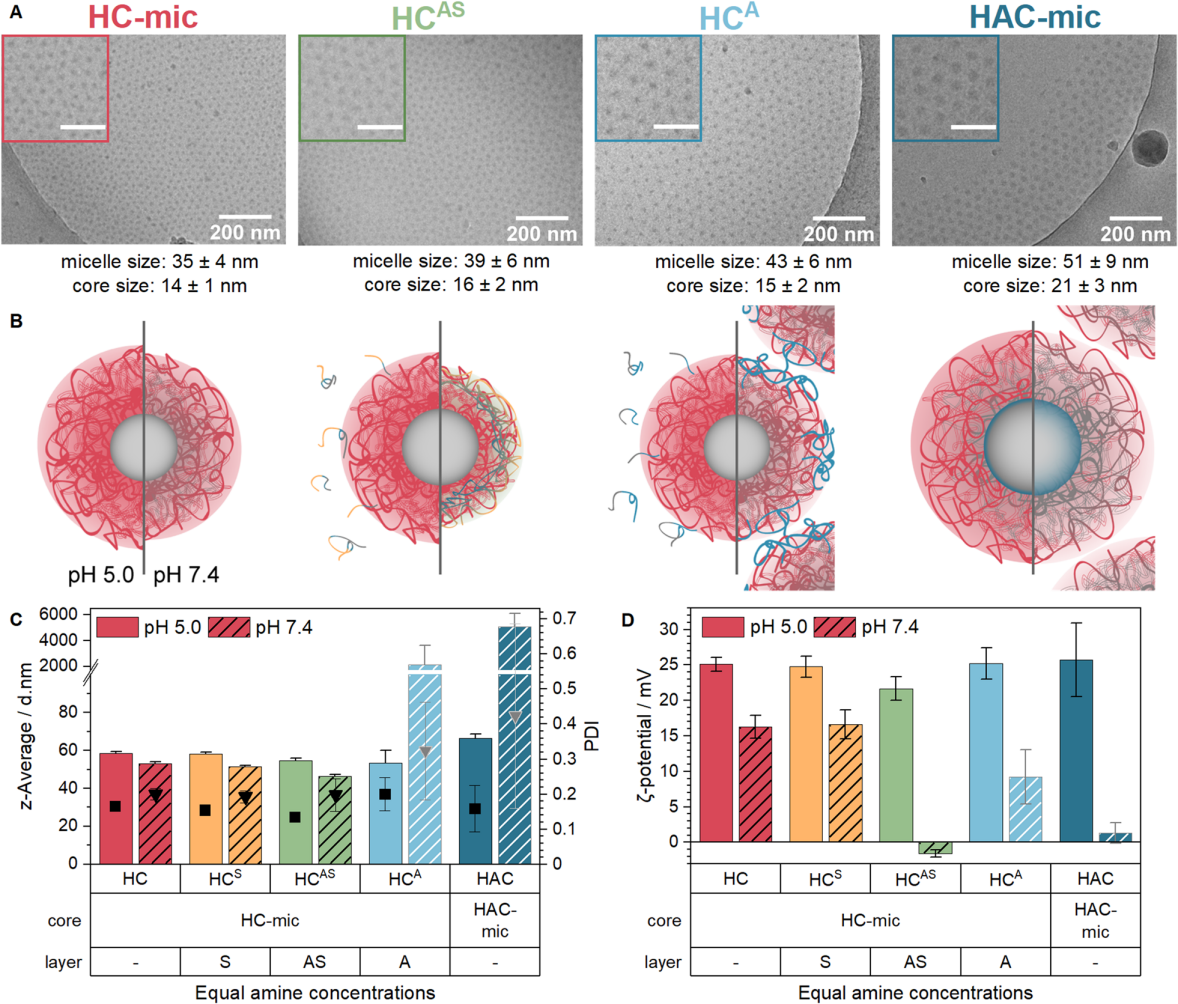
Characterization of (layered) micelles at different pH-values. **A** Cryo-TEM and images of HAC-mic, HC-mic and HC-mic layered with PAA (A) or P(NAM-*b*-AA) (AS) at pH 5. Scale bars in insets represent 100 nm. Micelles in images were analyzed using ImageJ as described in Additional file 1. **B** + **C** DLS (**B**) and ELS (**C**) measurements of (layered) micelles at equal amine content in acetate-HEPES buffer of pH 5.0 or 7.4. Values represent mean ± SD (n = 2) of z-average (columns), PDI (triangles) or ζ-potential (columns). Bright/gray shades indicate formation of aggregates. **D** Schematic representation of (layered) micelles at pH 5.0 and 7.4 (left and right side, respectively)

The effect of a post-assembly addition of shielding to the HC-mic was investigated by mixing the HC-mic solution with shielding polymer solutions 3 + 1 (*v/v*) resulting in carboxy to amine (COOH/NH) ratios of 0.5 for the HC^A^ and HC^AS^ assemblies, which was similar to the HAC-mic. The assembly of HC-mic and PNAM (S—stealth, HC^S^) was prepared with a PNAM/P*n*BA molar ratio of 1.0. To investigate the behavior of the (layered) micelles at pH-values relevant for biological studies, the different assemblies were tested in 100 mM acetate-HEPES buffer of pH 5.0 or pH 7.4. At pH 5, cryo-TEM, DLS and ELS measurements showed no considerable differences regarding the size, morphology or the surface charge of the assemblies compared to the naked HC-mic (Fig. 3). Due to the measurement of the hydrodynamic diameter by DLS, the assemblies appeared to be slightly larger (55–66 nm) than in the cryo-TEM images (35–51 nm) but both methods provided comparable tendencies: The HAC micelles were slightly larger than the HC micelles, which can be attributed to the presence of the additional third PAA block. This partially uncharged anionic PAA block in the HAC-mic at pH 5.0 might form intramicellar interpolyelectrolyte complexes (im-IPECs) with protonated PDMAEMA or collapse to the micelle core [61], leading to a slightly increased size of the micelle core (21 ± 3 nm) in the cryo-TEM images compared to the HC-mic (14 ± 1 nm). With ζ-potentials in the order of 24 mV, all assemblies showed strong positive surface charges.

However, when DLS measurements were performed at pH 7.4, the hydrodynamic diameter decreased by about 7 ± 2 nm for HC-mic, HC^S^ and HC^AS^. By contrast, HAC-mic and HC^A^ formed large aggregates and turbid suspensions, which could be due to neutralization of PDMAEAm by PAA (Fig. 2). Moreover, the ζ-potential of all assemblies was decreased when measured in 100 mM acetate-HEPES buffer of pH 7.4, with the greatest difference being observed for the anionic polymer-containing assemblies HAC-mic, HC^A^ and HC^AS^. Interestingly, HC^AS^ exhibited a ζ-potential close to zero and comparable to the HAC-mic, but still formed stable and defined nanostructures instead of aggregates at pH 7.4. This indicates a beneficial contribution of the additional hydrophilic PNAM block to the micelle stability, in particular at neutral pH-values.

### Polyplex formation and characterization

Since the micelles were designed for the purpose of transporting genetic material into cells, their interaction with genetic material was investigated with the combined ethidium bromide binding and heparin release assay (EBA, HRA) as described previously [53]. The assay uses the increase in the relative fluorescence intensity (rFI) of ethidium bromide (EtBr) upon (re)intercalation into the pDNA as a fluorescence indicator for unbound pDNA. A decreasing rFI relates to displacement of EtBr from the pDNA due to the binding of polymers. The polysaccharide heparin was used to investigate the stability of the polymer–pDNA complexes (polyplexes) against polyanions outside the cells, since it competes with the pDNA for the binding to the polymers. As a starting point, an N*/P ratio (active amines of the polymer to phosphates of the pDNA) of 30 was chosen. To investigate the influence of shielding on the obtained polyplexes, the respective shielding polymers were added post-polyplex formation at an COOH/NH-ratio of 0.5 (HC^A^, HC^AS^) or at a PNAM/P*n*BA molar ratio of 1.0 (HC^S^). To increase biocompatibility and avoid aggregation of HAC-mic and HC^A^ but still enable micelle-shielding interaction, a less strong buffer system was used for this and all further assays involving complexes of pDNA and polymer (polyplexes). The buffer contained 20 mM HEPES and 5% (w/v) glucose at pH 7.4 (HBG-buffer), resulting in pH-values of the polyplex solutions of pH 6.3 (HAC-mic) and 7.2 (remaining assemblies), which will be adjusted to pH 7.4 upon 1:10 dilution of the polyplexes with growth medium of pH 7.4 for cell treatment.

**Fig. 4:**
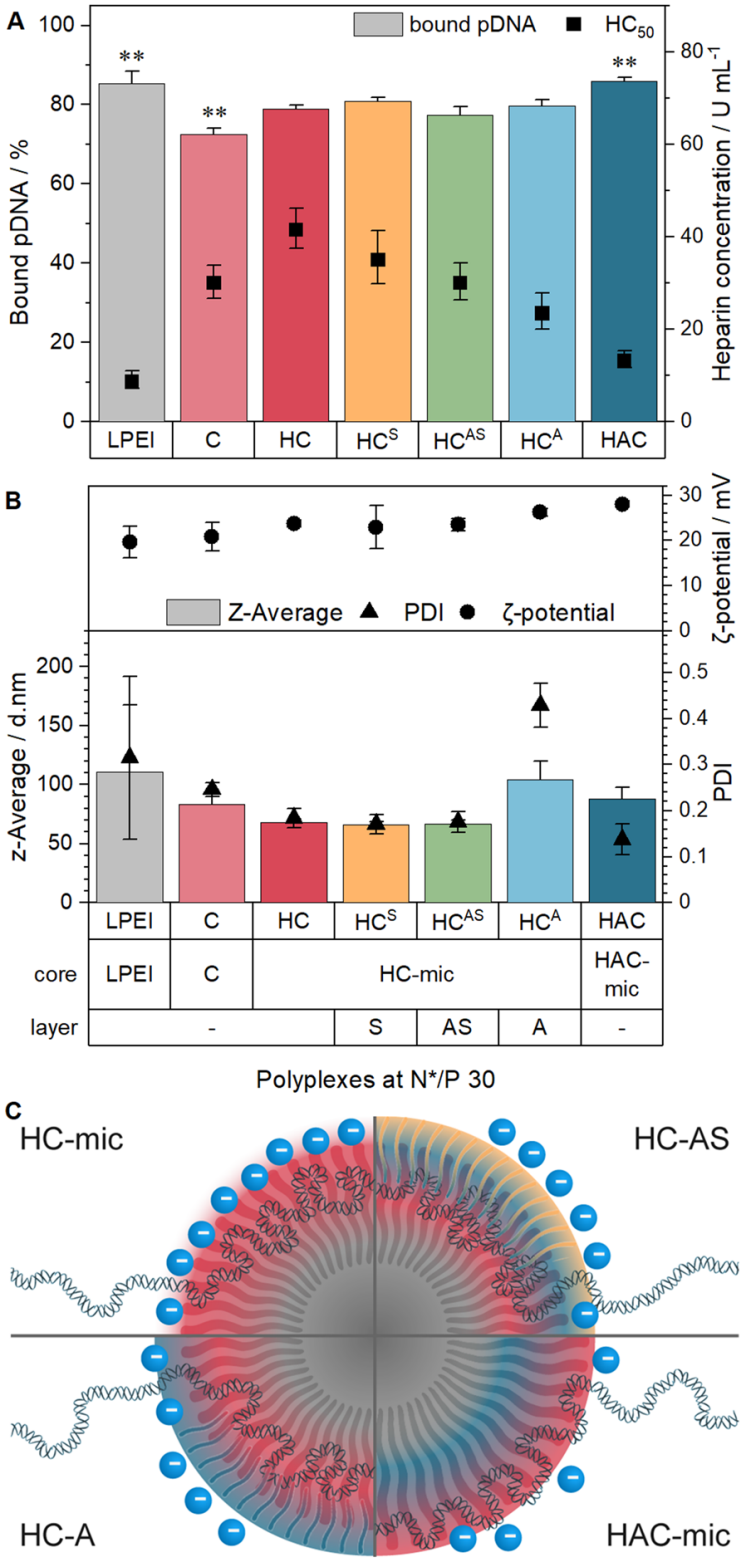
Complex formation with pDNA and stability tests. **A** EBA and HRA of (layered) polyplexes at N*/P 30 in HBG buffer. Columns represent EBA results as mean ± SD (n = 3). Dots represent HRA results with mean ± CI (confidence interval, n = 3). HC_50_ indicates heparin concentration required to release 50% of pDNA and was calculated with logistic fit functions (see Additional file 1). **B** DLS and ELS measurement of (layered) polyplexes at N*/P 30 in HBG buffer. Values represent mean ± SD (n = 2) of z-average (columns), PDI (triangles) or ζ-potential (dots). **C** Schematic representation of interactions between micelles, pDNA, shielding polymers and heparin (blue dots)

The results showed a high proportion of bound pDNA for all assemblies from 73 ± 2% (PDMAEAm, C) to 86 ± 1% (HAC-mic) being comparable to the commercial control linear poly(ethylene imine) (LPEI, 85 ± 3%, Fig. 4A). While slight increases were observed for the introduction of a hydrophobic core to the cationic PDMAEAm homopolymer (HC-mic: 79 ± 1%) and for the addition of the anionic block (HAC-mic), the addition of the shielding polymers did not change the proportion of bound pDNA compared to the naked micelle (HC-mic). Regarding the polyplex stability, the HC-mic required the highest concentration of heparin to release 50% of the pDNA, (HC_50_: 41.6 U mL^−1^) indicating a strong polymer–pDNA-interaction. The addition of the shielding polymers led to decreased HC_50_-values for the layered micelle assemblies compared to the naked micelle (HC-mic) with the HC^A^ showing the lowest values (23.5 U mL^−1^). However, they were still higher than the HAC-mic (13.2 U mL^−1^), which was comparable to LPEI (8.7 U mL^−1^), and therefore represents a good candidate for transfection efficiency assays.

The decreased polyplex stability of the HAC-mic can be caused by the covalent connection of the anionic PAA with the cationic PDMAEAm blocks, effectively reducing the amount of excess positively charged amines available after polyplex formation (N*/P ratio). Therefore, a lower amount of heparin can be trapped by excess cationic charges (see also Fig. 4C). By contrast, the polyplex in the HC^A^ assembly was formed in absence of PAA, which can therefore interact only with the remaining, free positively charged amines not occupied by the phosphate groups of the pDNA. Since the negatively charged heparin is repelled by the negatively charged PAA, increased concentrations of heparin were required to release the same amount of pDNA as the HAC-mic.

To further investigate the polyplex properties, DLS and ELS measurements were performed with (layered) polyplexes at N*/P 30 to determine the hydrodynamic diameter of the assemblies. Compared to the assemblies without pDNA in HBG buffer (Additional file 1: Figure S10), the formation of polyplexes led to slightly increased sizes (Fig. 4B), as observed in other studies before [23, 24, 62]. All polyplexes were slightly below 100 nm in diameter being optimal for endocytotic uptake [63]. The HC-mic and the HC^S^ and HC^AS^ assemblies exhibited the smallest polyplexes (≈ 70 nm), whereas the other assemblies showed sizes of 90 to 100 nm. Regarding the HC^A^ assembly, two distinct peaks were observed in the intensity plot (Additional file 1: Figure S8). Together with the increased PDI values, this could indicate the aggregation of several micelles due to the strong attraction between the positively charged shell of the HC-mic and the negatively charged shielding polymer PAA. Regarding the electrical potential, all assemblies containing micelles exhibited similar ζ-potentials of about 25 mV, which was slightly higher compared to the homopolymer polyplexes (C, 21 ± 3 mV) and could be explained with an increased charge density in the micellar corona [24].

### Cytotoxicity

As polycations are known for their interaction with cell membranes, different cytotoxicity assays were performed: (i) the PrestoBlue assay determining the metabolic activity, (ii) the LDH release assay determining the membrane integrity and (iii) flow cytometry determining the cell viability due to their appearance in the FSC/SSC plot. For all assays, the cells were incubated with the above described (layered) polyplexes.

**Fig. 5:**
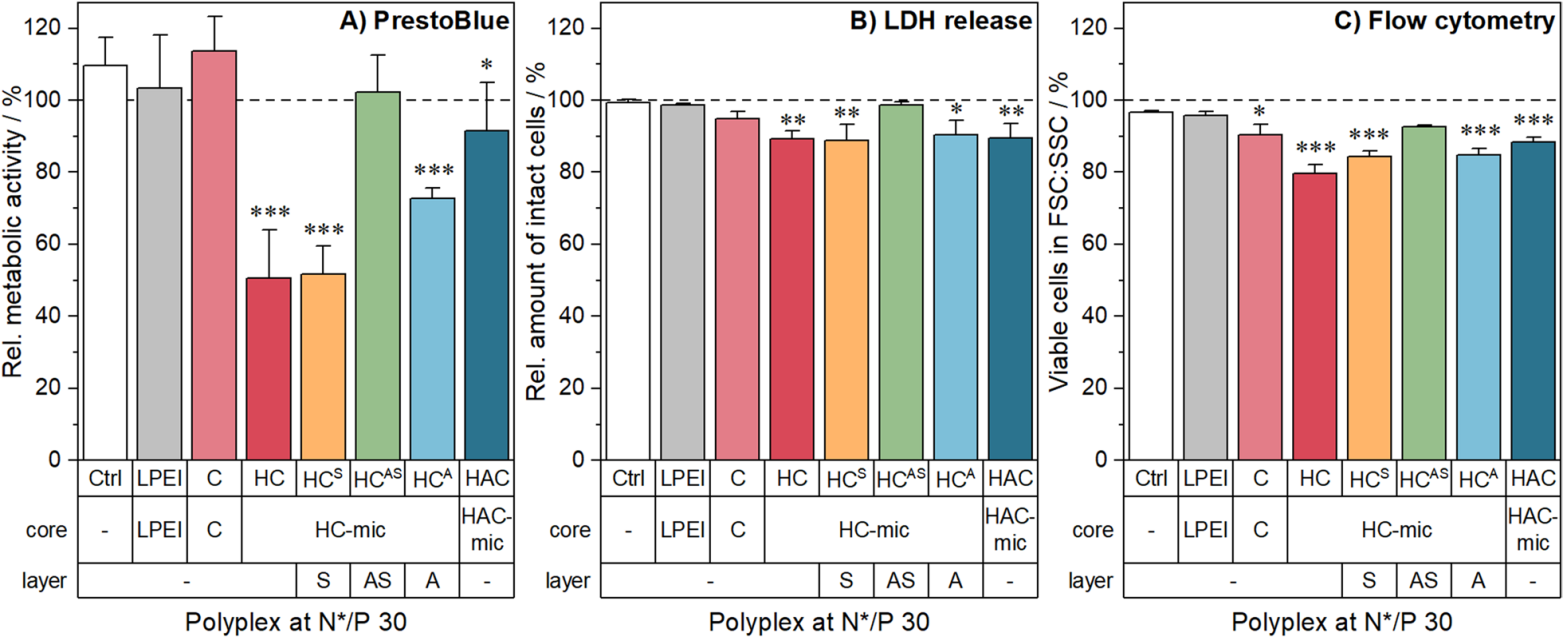
Toxicity of polyplexes in HEK293T cells. The cells were treated with (layered) polyplexes at N*/P 30 for 24 h. Incubation with pDNA only was used as control. Subsequently, the viability of the cells was investigated using **A** the PrestoBlue assay for metabolic activity, **B** the LDH release assay, or **C** flow cytometry with gating for viable cells in the FSC/SSC plot. Cells treated with pDNA only served as control. Values represent mean ± SD (n = 3). */**/***Significant differences to control (*p* < 0.05/0.01/0.001)

The evaluation of the metabolic activity revealed differences between the assemblies. Polyplexes of the HC-mic and the HC^S^ assembly caused the highest reduction in cell viability (≈ 50%), whereas cells incubated with the PDMAEAm polyplexes (C) showed no cytotoxicity (Fig. 5A). This indicates that the hydrophobic block and the micellar structure of the HC-mic contributed to cytotoxic effects, which have been also observed with other hydrophobic-cationic micelle systems and can be explained by a high local concentration of cationic moieties [23]. The addition of anionic polymer, either post-polyplex formation (HC^A^) or within the micelle (HAC-mic), showed medium to low toxicity, while the combination with the stealth polymer PNAM (HC^AS^) eliminated the cytotoxic effect. Layering with only PNAM in the HC^S^ assembly did not reduce cytotoxicity, since this assembly lacks an anionic counterpart for ionic interaction with the micelle. Furthermore, the HAC-mic also showed toxicity alleviating effects compared to the HC-mic in concentration dependent studies without pDNA in L-929 cells (Additional file 1: Figure S12A), which might be due to the decreased degree of positive charges in the HAC-mic.

In contrast to the PrestoBlue assay, there were only slight differences between the different polyplex assemblies in the LDH-release and flow cytometry assays. All assemblies led to viabilities above 80% (Fig. 5B, C). This indicates only slight or no influence of the polymers on the cell membrane and morphology, which is supported by hemolysis and aggregation assays with human erythrocytes and in microscopic investigations of HEK293T cells (Additional file 1: Figures S12C, D, S13). However, cells incubated with the HC^AS^ assembly or the HAC-mic exhibited the highest viabilities and, therefore, represent promising candidates for further studies.

### EGFP expression with (layered) micelles

**Fig. 6:**
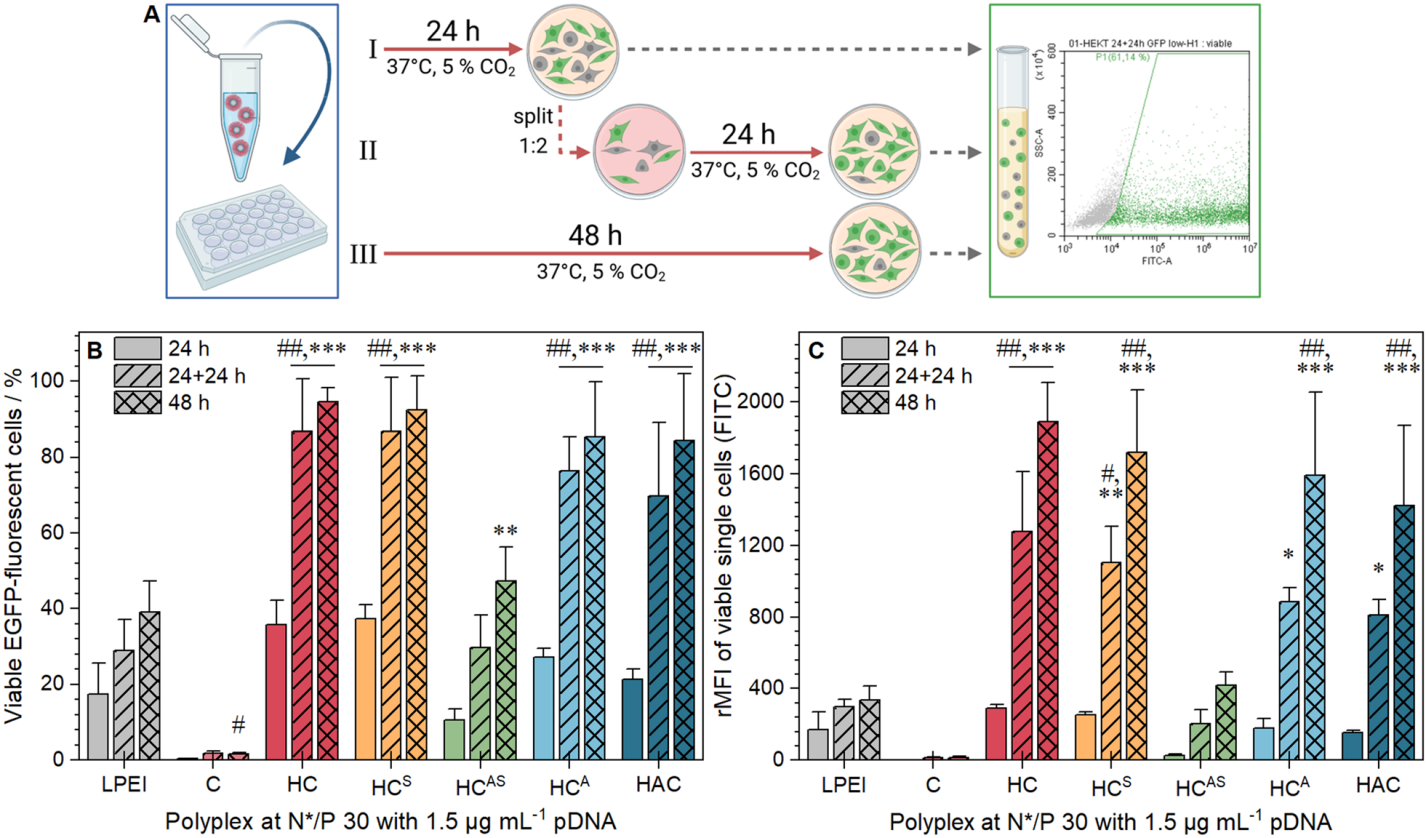
Transfection efficiency of polymers in HEK293T cells. EGFP expression of viable cells was analyzed via flow cytometry. **A** Schematic representation of the incubation method (created with BioRender.com). Cells were incubated with (layered) polyplexes of mEGFP-N1 pDNA and polymers at N*/P 30 in growth medium for 24 h (I), for 24 h followed by splitting of cells and medium and further incubation for 24 h (II), or for 48 h (III). Values represent mean ± SD (n = 3) of (**B**) viable, single EGFP positive cells (**C**) rMFI of all viable single cells relative to cells treated with polyplexes of pKMyc pDNA and polymers. ^##^Significant difference to HC-mic at respective time points (*p* < 0.001), */**/***significant difference to same polymer after 24 h (*p* < 0.05/0.01/0.001)

Since all polymers exhibited high interaction with pDNA and led to low to moderate cytotoxicity, their transfection efficiency (amount of EGFP expressing cells) was investigated. For a better understanding of possible shielding effects, different incubation periods were examined. Therefore, HEK293T cells were incubated with the (layered) polyplexes at N*/P 30 for 24 h, 48 h or for 24 h followed by 1:2 splitting of the cells with fresh medium and an additional 24 h observation period (Fig. 6A). Subsequently, flow cytometry was used to determine the amount and relative mean fluorescence intensity (rMFI) of EGFP expressing cells. The results showed different EGFP expression for all assemblies in serum-containing media with slightly higher efficiencies than LPEI and mostly consistent within different assembly batches (Fig. 6, Additional file 1: Figure S16). A fourfold increase in transfection efficiency was observed when the incubation time (48 h) or the observation time (24 + 24 h) were extended, leading to transfection efficiencies of up to 95% viable EGFP positive cells with no changes in cytotoxicity (Additional file 1: Figure S12B). After 48 h, the homopolymer PDMAEAm resulted in nearly no transfected cells, whereas the HC-mic showed the highest transfection efficiency (95 ± 4% viable EGFP positive cells). The layering with PNAM alone (HC^S^) did not influence the effect of the HC-mic, but in combination with the anionic block (HC^AS^) the transfection efficiency was significantly reduced (47 ± 9%), but was still comparable with the commercial control LPEI. The two PAA containing assemblies without PNAM (HAC-mic and HC^A^) were comparable to the HC-mic (85 ± 14 and 85 ± 18%, respectively).

Furthermore, LPEI could only reach these values when the pDNA concentration was increased to 3.0 µg mL^−1^ and the polymer concentration remained the same (Additional file 1: Figure S16), whereas the transfection efficiencies of the assemblies did not change. This indicates a higher efficacy of these systems, requiring only half of the pDNA to achieve similar transfection rates as LPEI. The reason might be seen in the differences between polyplexes of micelles and pDNA compared to those of homopolymers with pDNA, leading to increased stability and the preservation of the pDNA structure as shown by Tan and coworkers [24]. Although the HC-mic showed higher efficiency than the HAC-mic under these conditions, it is worth noticing, that HAC-mic outperformed the HC-mic at higher polymer concentrations due to cytotoxicity issues (Additional file 1: Figure S17).

### Transfection mechanism of (layered) micelles

**Fig. 7:**
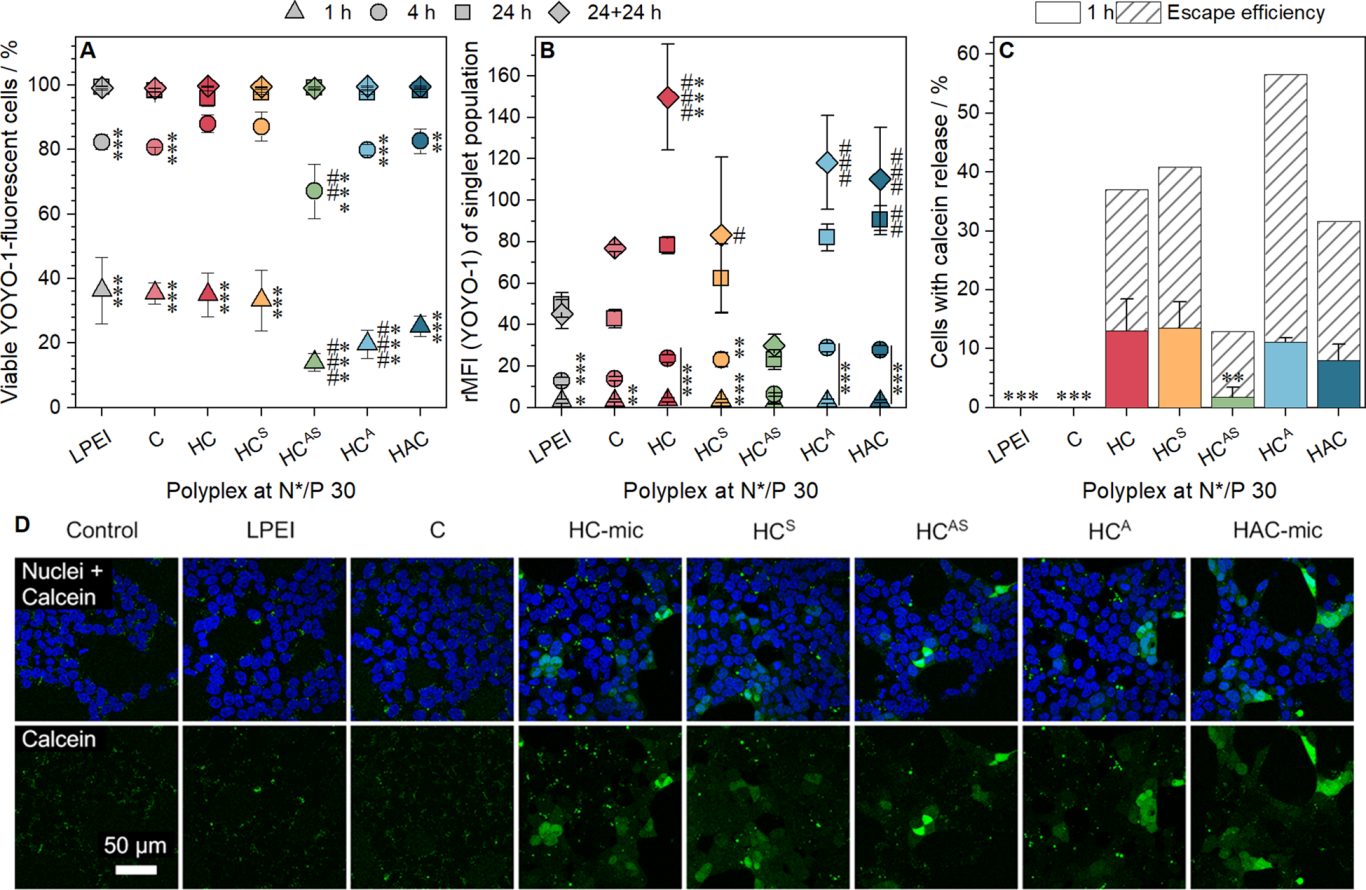
Investigation of the gene delivery process in HEK293T cells. **A** + **B** Cellular uptake of polyplexes in HEK293T cells. Cells were incubated with (layered) polyplexes of polymers and YOYO-1-labeled pDNA at N*/P 30 for different time periods and analyzed via flow cytometry. Cells incubated with labeled pDNA served as control (rMFI = 1). Values represent mean ± SD of **A** viable, single YOYO-1 positive cells and **B** rFMI of all viable, single cells relative to cells treated with YOYO-1 labeled pDNA only. ^#/##/###^Significant difference to LPEI at respective time point (*p* < 0.05/0.01/0.001), */**/***significant difference to same polymer after 24 h (*p* < 0.05/0.01/0.001). **C** + **D** Endosomal escape was analyzed via CLSM following simultaneous incubation with the non-permeable dye calcein (green) and (layered) polyplexes of polymers and pDNA at N*/P 30 (not stained) for 1 h. The cell nuclei were stained with Hoechst 33342 (blue). Values in (**C**) were obtained by image analysis of all acquired images using ImageJ and represent mean ± SD (n = 3) of the number of cells with extensive green fluorescence relative to the total amount of cells (Hoechst-stained nuclei). **/***Significant difference to HC-mic (*p* < 0.01/0.001). Shaded columns represent the proportion of cells with calcein release divided by the proportion of YOYO-1 positive cells at the same time point (in percent). Green dots in (**D**) indicate calcein within cellular compartments, whereas a diffuse green fluorescence pattern indicates calcein released to the cytosol

To gain an in-depth look into the transfection mechanism, the (layered) polyplexes were investigated regarding their performance at two crucial steps of the transfection process: (i) polyplex uptake and (ii) endosomal escape. For the polyplex uptake, the HEK293T cells were incubated with (layered) polyplexes of YOYO-1 labeled pDNA and polymers at N*/P 30 in serum-containing media for different time periods, before they were measured and analyzed via flow cytometry regarding the amount of YOYO-1 positive cells and the relative MFI (rMFI) of viable single cells (Fig. 7A, B). All assemblies exhibited a time-dependent increase of the uptake in nearly all cells after 24 h. Following 1 h of incubation, 14 to 36% of the cells were YOYO-1 positive with comparable rMFI values. Although, all cells showed polyplex uptake after 24 h, the anionic polymer containing assemblies led to slightly increased rMFI values compared to the pure cationic assemblies (HAC/HC^A^: 86 *vs.* HC/HC^S^: 70, *p* = 1.000). The HC^AS^ assembly exhibited the lowest proportion of YOYO-1 positive cells and rMFI values. This could be due to the “stealth-dilemma” or due to a decreased aggregation number of micelles within one polyplex as it was shown for PEG-*b*-PDMAEMA-*b*-P*n*BMA micelles [62]. Interestingly, the rMFI values for the successful assemblies (HC, HC^A^, HC^S^, HAC) increased although the cells and medium were split after 24 h and the observation time was increased (24 + 24 h, Fig. 6AII). This could point towards an interaction between these polymers and either cells or the culture vessel which is not disturbed by trypsinization.

The uptake of polyplexes following incubation of 1 h was further confirmed by CLSM (Additional file 1: Figure S20) showing a similar trend for the number of polyplexes per cell as observed via flow cytometry. By staining the plasma membrane and membrane originating organelles with CellMask Deep Red Plasma membrane stain, a colocalization analysis of polyplexes could be performed, which revealed the presence of about 10% free polyplexes for all treatments. Therefore, the second crucial cellular hurdle for transfection efficiency, the endosomal escape, was investigated using calcein as a non-permeable fluorescence dye leading to (i) a dotted pattern inside the cells following uptake via endocytosis or (ii) a diffuse fluorescence pattern upon its release into the cytosol if the endosomal membrane was disrupted, e.g., by a polymer. The HEK293T cells were incubated as described above with (layered) polyplexes at N*/P 30 and calcein. Following washing of the cells and staining of nuclei with Hoechst 33342, images were acquired by CLSM (Fig. 7D). The quantification of the number of cells showing calcein release was performed with ImageJ (see Additional file 1: Methods), and revealed that only the micellar assemblies displayed remarkable calcein release (Fig. 7C). The absence of calcein release by LPEI and PDMAEMA could point towards different endosomal escape mechanisms for linear polymers and micellar systems. As an example, different types of calcein leakage from vesicles (graded, all-or-none) have already been reported for detergents [64]. Moreover, differences were also observed for the fluorescence pattern of the polyplexes in the CLSM uptake study (Additional file 1: Figure S21, weak fluorescence of linear polymers vs. bright, large spots for micellar systems), which could be an indication of different amounts of YOYO-1 labeled pDNA. However, the exact mechanism and quantification of the endosomal escape remain to be elucidated. All in all, the values of the micelles correlated well with those observed for transfection efficiency, with HC-mic and HC^S^ leading to the highest (13 ± 5 and 14 ± 4% cells, respectively) and HC^AS^ exhibiting the lowest calcein release (1.8 ± 1.6% cells). Calculating the escape efficiency [proportion of cells positive for calcein release (Fig. 7C) normalized to the proportion of YOYO-1 positive cells (Fig. 7A, 1 h)] demonstrated a superior escape efficiency for the HC^A^ assembly. This could be due to an enhanced surface-accessibility of the PAA outside the micelle compared to the HAC-mic leading to a pH-dependent unmasking of cationic charges, and to an increased concentration of molecules which could also be beneficial for endosomal escape.

## Conclusion

Since cationic polymers for non-viral gene delivery are known to be cytotoxic and immune active, shielding polymers with anionic or hydrophilic moieties are being investigated, either covalently bound within block copolymers forming micelles or as a post-formulation addition to nanoparticles and homopolymer polyplexes. In this study, the post-formulation addition of three different shielding polymers, PAA, PNAM and P(NAM-*b*-AA), to a cationic micelle of P(*n*BA-*b*-DMAEAm) (HC-mic) was compared to a triblock terpolymer micelle containing the anionic PAA as the middle block (HAC-mic). With the exception of the commercial PAA, the polymers were synthesized via RAFT polymerization, resulting in well-defined homo- and block copolymers. The assembled micelles were small (around 50 nm) with narrow size distributions, favored for biological investigations. Layered micelles were obtained by mixing the shielding polymers with the HC-mic.

**Fig. 8:**
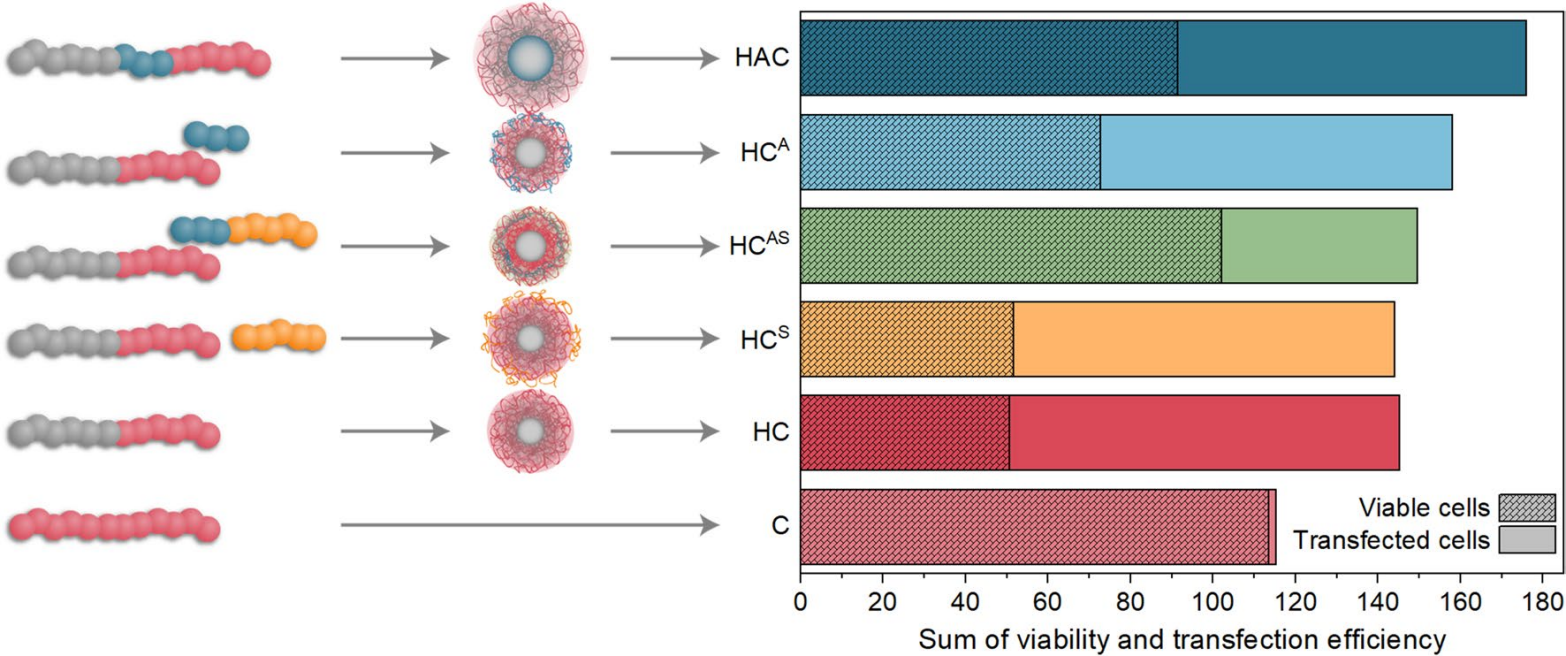
Summary of the influence of different shielding polymers on the HC-mic’s performance. Addition of viable EGFP-positive HEK293T cells following incubation with (layered) polyplexes for 48 h in % to the relative amount of metabolically active HEK293T cells following incubation with (layered) polyplexes for 24 h in %. Scheme on the left indicates the composition of the polyplexes with one sphere representing about 14 repeating units of the polymer

The assemblies were characterized regarding their performance at crucial key steps for gene delivery, such as interaction with pDNA, cytotoxicity, transfection efficiency, polyplex uptake, and endosomal escape. The naked HC-mic formed the most stable complexes with pDNA and exhibited increased transfection efficiency (95% transfected cells following prolonged incubation) that was even twice as efficient as the commercial control LPEI. However, only moderate cell viabilities were observed. Among the different shielding polymers, the anionic PAA could alleviate the cytotoxic effects with nearly no loss in transfection efficiency, irrespective of whether it was part of the parent micelle (HAC-mic) or applied as a shielding polymer (HC^A^). Nevertheless, there were slight differences between both assemblies with the HAC-mic showing slightly less stable polyplex formation and less efficient endosomal escape compared to HC^A^. This indicates a stronger influence of PAA on the HC-mic’s performance when covalently incorporated inside the micelle than when electrostatically interacting with the micelle corona. Still, the HAC-mic convinced with the best ratio of viability to transfection efficiency (Fig. 8).

The highest shielding effect was observed for the combination of anionic and hydrophilic polymer (HC^AS^). It formed moderately stable polyplexes with pDNA and preserved the micellar structure even at neutral ζ-potentials but reached only 50% of the HC-mic’s transfection efficiency, which still resulted in 40% transfected cells. Remarkably, HC^AS^ was able to completely eliminate cytotoxic effects which can be attributed to a decreased membrane interaction, which was also indicated by a severely reduced uptake into the cells. In combination with suitable ligands, this shielding block copolymer could therefore be applied for targeted gene delivery in vivo additionally offering the benefits of simple synthesis and flexible combination with different core micelles. Both, the HAC-mic and the HC^AS^ system led to the highest cell viability with still good transfection efficiency and will be investigated in further studies. Moreover, these findings illustrate the dependence of the system’s performance on the precise molecular arrangement of the polymer blocks. Even the incorporation of anionic polymer blocks alone greatly improved the performance of the HC-mic by reducing the cytotoxic effect of the naked cationic micelles without mitigating their efficiency. The addition of stealth polymers further improved their toxicity profile. Therefore, layered micelles represent an efficient design principle for gene transfer due to the synthesis of diblock copolymers being less challenging than multiblock copolymers, and the possibility to vary micelle and layer in a modular fashion allowing adjustments for further applications. The toxicity and side effects of transfection can thus be efficiently and elegantly circumvented.

## Supplementary Information

**Additional file 1.** Materials, additional methods and supplementary figures.

## Declarations

### Availability of data and materials

The datasets used and/or analyzed during the current study are available from the corresponding author on reasonable request. Additional information available: Material, additional methods and results, Additional Figures S1–S22, Additional Tables S1–S8 (PDF).

## References

[1] Zhang P, Wagner E (2017). History of polymeric gene delivery systems. Top Curr Chem (Cham).

[2] Wahane A, Waghmode A, Kapphahn A, Dhuri K, Gupta A, Bahal R (2020). Role of lipid-based and polymer-based non-viral vectors in nucleic acid delivery for next-generation gene therapy. Molecules.

[3] Campos EVR, Pereira AES, de Oliveira JL, Carvalho LB, Guilger-Casagrande M, de Lima R, Fraceto LF (2020). How can nanotechnology help to combat COVID-19? Opportunities and urgent need. J Nanobiotechnol.

[4] Tang Z, Zhang X, Shu Y, Guo M, Zhang H, Tao W (2021). Insights from nanotechnology in COVID-19 treatment. Nano Today.

[5] Tang Z, Kong N, Zhang X, Liu Y, Hu P, Mou S, Liljestrom P, Shi J, Tan W, Kim JS (2020). A materials-science perspective on tackling COVID-19. Nat Rev Mater.

[6] Jhaveri AM, Torchilin VP (2014). Multifunctional polymeric micelles for delivery of drugs and siRNA. Front Pharmacol.

[7] Durymanov M, Reineke J (2018). Non-viral delivery of nucleic acids: insight into mechanisms of overcoming intracellular barriers. Front Pharmacol.

[8] Gregory A, Stenzel MH (2012). Complex polymer architectures via RAFT polymerization: from fundamental process to extending the scope using click chemistry and nature's building blocks. Prog Polym Sci.

[9] Matyjaszewski K (2012). Atom transfer radical polymerization (ATRP): current status and future perspectives. Macromolecules.

[10] Siegwart DJ, Oh JK, Matyjaszewski K (2012). ATRP in the design of functional materials for biomedical applications. Prog Polym Sci.

[11] Moad G (2017). RAFT polymerization to form stimuli-responsive polymers. Polym Chem.

[12] Moad CL, Moad G (2021). Fundamentals of reversible addition–fragmentation chain transfer (RAFT). Chem Teach Int.

[13] Ahmed M, Narain R (2013). Progress of RAFT based polymers in gene delivery. Prog Polym Sci.

[14] Cabral H, Miyata K, Osada K, Kataoka K (2018). Block copolymer micelles in nanomedicine applications. Chem Rev.

[15] Oerlemans C, Bult W, Bos M, Storm G, Nijsen JF, Hennink WE (2010). Polymeric micelles in anticancer therapy: targeting, imaging and triggered release. Pharm Res.

[16] Letchford K, Burt H (2007). A review of the formation and classification of amphiphilic block copolymer nanoparticulate structures: micelles, nanospheres, nanocapsules and polymersomes. Eur J Pharm Biopharm.

[17] Mane SR, Sathyan A, Shunmugam R (2020). Biomedical applications of pH-responsive amphiphilic polymer nanoassemblies. ACS Appl Nano Mater.

[18] Gao D, Guo X, Zhang X, Chen S, Wang Y, Chen T, Huang G, Gao Y, Tian Z, Yang Z (2020). Multifunctional phototheranostic nanomedicine for cancer imaging and treatment. Mater Today Bio.

[19] Wang H, Ding S, Zhang Z, Wang L, You Y (2019). Cationic micelle: a promising nanocarrier for gene delivery with high transfection efficiency. J Gene Med.

[20] Navarro G, Pan J, Torchilin VP (2015). Micelle-like nanoparticles as carriers for DNA and siRNA. Mol Pharm.

[21] Uchida S, Kataoka K (2019). Design concepts of polyplex micelles for in vivo therapeutic delivery of plasmid DNA and messenger RNA. J Biomed Mater Res A.

[22] Yousefpour Marzbali M, Yari Khosroushahi A (2017). Polymeric micelles as mighty nanocarriers for cancer gene therapy: a review. Cancer Chemother Pharmacol.

[23] Richter F, Mapfumo P, Martin L, Solomun JI, Hausig F, Frietsch JJ, Ernst T, Hoeppener S, Brendel JC, Traeger A (2021). Improved gene delivery to K-562 leukemia cells by lipoic acid modified block copolymer micelles. J Nanobiotechnol.

[24] Tan Z, Jiang Y, Zhang W, Karls L, Lodge TP, Reineke TM (2019). Polycation architecture and assembly direct successful gene delivery: micelleplexes outperform polyplexes via optimal DNA packaging. J Am Chem Soc.

[25] Convertine AJ, Benoit DS, Duvall CL, Hoffman AS, Stayton PS (2009). Development of a novel endosomolytic diblock copolymer for siRNA delivery. J Control Release.

[26] Liu ZH, Zhang ZY, Zhou CR, Jiao YP (2010). Hydrophobic modifications of cationic polymers for gene delivery. Prog Polym Sci.

[27] Monnery BD, Wright M, Cavill R, Hoogenboom R, Shaunak S, Steinke JHG, Thanou M (2017). Cytotoxicity of polycations: relationship of molecular weight and the hydrolytic theory of the mechanism of toxicity. Int J Pharm.

[28] van de Wetering P, Cherng J-Y, Talsma H, Hennink WE (1997). Relation between transfection efficiency and cytotoxicity of poly(2-(dimethylamino)ethyl methacrylate)/plasmid complexes. J Control Release.

[29] Lv H, Zhang S, Wang B, Cui S, Yan J (2006). Toxicity of cationic lipids and cationic polymers in gene delivery. J Control Release.

[30] Pack DW, Hoffman AS, Pun S, Stayton PS (2005). Design and development of polymers for gene delivery. Nat Rev Drug Discov.

[31] Hu CM, Fang RH, Luk BT, Zhang L (2014). Polymeric nanotherapeutics: clinical development and advances in stealth functionalization strategies. Nanoscale.

[32] Yang JL, Zhang XC, Liu C, Wang Z, Deng LF, Feng C, Tao W, Xu XY, Cui WG (2021). Biologically modified nanoparticles as theranostic bionanomaterials. Prog Mater Sci.

[33] Fang Y, Xue J, Gao S, Lu A, Yang D, Jiang H, He Y, Shi K (2017). Cleavable PEGylation: a strategy for overcoming the "PEG dilemma" in efficient drug delivery. Drug Deliv.

[34] Mishra S, Webster P, Davis ME (2004). PEGylation significantly affects cellular uptake and intracellular trafficking of non-viral gene delivery particles. Eur J Cell Biol.

[35] Li B, Yuan Z, Hung HC, Ma J, Jain P, Tsao C, Xie J, Zhang P, Lin X, Wu K, Jiang S (2018). Revealing the immunogenic risk of polymers. Angew Chem Int Ed Engl.

[36] Peeler DJ, Sellers DL, Pun SH (2019). pH-sensitive polymers as dynamic mediators of barriers to nucleic acid delivery. Bioconjug Chem.

[37] Yang Q, Jacobs TM, McCallen JD, Moore DT, Huckaby JT, Edelstein JN, Lai SK (2016). Analysis of pre-existing IgG and IgM antibodies against polyethylene glycol (PEG) in the general population. Anal Chem.

[38] Kierstead PH, Okochi H, Venditto VJ, Chuong TC, Kivimae S, Frechet JMJ, Szoka FC (2015). The effect of polymer backbone chemistry on the induction of the accelerated blood clearance in polymer modified liposomes. J Control Release.

[39] Ishihara T, Maeda T, Sakamoto H, Takasaki N, Shigyo M, Ishida T, Kiwada H, Mizushima Y, Mizushima T (2010). Evasion of the accelerated blood clearance phenomenon by coating of nanoparticles with various hydrophilic polymers. Biomacromol.

[40] Felber AE, Dufresne MH, Leroux JC (2012). pH-sensitive vesicles, polymeric micelles, and nanospheres prepared with polycarboxylates. Adv Drug Deliv Rev.

[41] Dewald I, Gensel J, Betthausen E, Borisov OV, Muller AH, Schacher FH, Fery A (2016). Splitting of surface-immobilized multicompartment micelles into clusters upon charge inversion. ACS Nano.

[42] Rinkenauer AC, Schallon A, Gunther U, Wagner M, Betthausen E, Schubert US, Schacher FH (2013). A paradigm change: efficient transfection of human leukemia cells by stimuli-responsive multicompartment micelles. ACS Nano.

[43] Synatschke CV, Nomoto T, Cabral H, Fortsch M, Toh K, Matsumoto Y, Miyazaki K, Hanisch A, Schacher FH, Kishimura A (2014). Multicompartment micelles with adjustable poly(ethylene glycol) shell for efficient in vivo photodynamic therapy. ACS Nano.

[44] Pergushov DV, Muller AH, Schacher FH (2012). Micellar interpolyelectrolyte complexes. Chem Soc Rev.

[45] Vergaro V, Scarlino F, Bellomo C, Rinaldi R, Vergara D, Maffia M, Baldassarre F, Giannelli G, Zhang X, Lvov YM, Leporatti S (2011). Drug-loaded polyelectrolyte microcapsules for sustained targeting of cancer cells. Adv Drug Deliv Rev.

[46] Finsinger D, Remy JS, Erbacher P, Koch C, Plank C (2000). Protective copolymers for nonviral gene vectors: synthesis, vector characterization and application in gene delivery. Gene Ther.

[47] Sethuraman VA, Na K, Bae YH (2006). pH-responsive sulfonamide/PEI system for tumor specific gene delivery: an in vitro study. Biomacromol.

[48] Nakamura N, Mochida Y, Toh K, Fukushima S, Cabral H, Anraku Y (2021). Effect of mixing ratio of oppositely charged block copolymers on polyion complex micelles for in vivo application. Polymers.

[49] Sanati S, Taghavi S, Abnous K, Taghdisi SM, Babaei M, Ramezani M, Alibolandi M (2021). Fabrication of anionic dextran-coated micelles for aptamer targeted delivery of camptothecin and survivin-shRNA to colon adenocarcinoma. Gene Ther.

[50] Yu L, Lin C, Zheng Z, Li Z, Wang X (2016). Self-assembly of pH-responsive biodegradable mixed micelles based on anionic and cationic polycarbonates for doxorubicin delivery. Colloids Surf B Biointerfaces.

[51] Colombani O, Ruppel M, Burkhardt M, Drechsler M, Schumacher M, Gradzielski M, Schweins R, Muller AHE (2007). Structure of micelles of poly(n-butyl acrylate)-block-poly (acrylic acid) diblock copolymers in aqueous solution. Macromolecules.

[52] Gardey E, Sobotta FH, Hoeppener S, Bruns T, Stallmach A, Brendel JC (2020). Influence of core cross-linking and shell composition of polymeric micelles on immune response and their interaction with human monocytes. Biomacromol.

[53] Richter F, Martin L, Leer K, Moek E, Hausig F, Brendel JC, Traeger A (2020). Tuning of endosomal escape and gene expression by functional groups, molecular weight and transfection medium: a structure-activity relationship study. J Mater Chem B.

[54] van de Wetering P, Moret EE, Schuurmans-Nieuwenbroek NM, van Steenbergen MJ, Hennink WE (1999). Structure-activity relationships of water-soluble cationic methacrylate/methacrylamide polymers for nonviral gene delivery. Bioconjug Chem.

[55] Martin L, Gody G, Perrier S (2015). Preparation of complex multiblock copolymers via aqueous RAFT polymerization at room temperature. Polym Chem.

[56] Schindelin J, Arganda-Carreras I, Frise E, Kaynig V, Longair M, Pietzsch T, Preibisch S, Rueden C, Saalfeld S, Schmid B (2012). Fiji: an open-source platform for biological-image analysis. Nat Methods.

[57] Gody G, Maschmeyer T, Zetterlund PB, Perrier S (2014). Exploitation of the degenerative transfer mechanism in RAFT polymerization for synthesis of polymer of high livingness at full monomer conversion. Macromolecules.

[58] Mellman I, Fuchs R, Helenius A (1986). Acidification of the endocytic and exocytic pathways. Annu Rev Biochem.

[59] Catrouillet S, Brendel JC, Larnaudie S, Barlow T, Jolliffe KA, Perrier S (2016). Tunable length of cyclic peptide-polymer conjugate self-assemblies in water. ACS Macro Lett.

[60] Huotari J, Helenius A (2011). Endosome maturation. EMBO J.

[61] Betthausen E, Drechsler M, Fortsch M, Schacher FH, Muller AHE (2011). Dual stimuli-responsive multicompartment micelles from triblock terpolymers with tunable hydrophilicity. Soft Matter.

[62] Jiang Y, Lodge TP, Reineke TM (2018). Packaging pDNA by polymeric ABC micelles simultaneously achieves colloidal stability and structural control. J Am Chem Soc.

[63] Bus T, Traeger A, Schubert US (2018). The great escape: how cationic polyplexes overcome the endosomal barrier. J Mater Chem B.

[64] Braun S, Pokorna S, Sachl R, Hof M, Heerklotz H, Hoernke M (2018). Biomembrane permeabilization: statistics of individual leakage events harmonize the interpretation of vesicle leakage. ACS Nano.

