# Supplementary material for: The impact of anionic polymers on gene delivery: how composition and assembly help evading the toxicity-efficiency dilemma
Source: J Nanobiotechnology. 2021 Sep 27;19:292. doi: 10.1186/s12951-021-00994-2 (PMC8477462; doi:10.1186/s12951-021-00994-2)
Supplement: Supplementary file 1 — Additional file 1. Materials, additional methods and supplementary figures. [file 12951_2021_994_MOESM1_ESM.pdf]

Additional file 1

The Impact of Anionic Polymers on Gene Delivery: How Composition and Assembly Help Evading the Toxicity-Efficiency Dilemma

Friederike Richter,[a ‡] *Katharina Leer,*[a ‡] *Liam Martin,*[a] *Prosper Mapfumo,*[a] *Jana I. Solomun,*[a] *Maren T. Kuchenbrod,*[a] *Stephanie Hoeppener,*[a,b] *Johannes C. Brendel,*[a,b] *Anja Traeger**[a,b]

[a]Laboratory of Organic and Macromolecular Chemistry (IOMC), Friedrich Schiller University Jena, Humboldtstrasse 10, 07743 Jena, Germany.

[b]Jena Center for Soft Matter (JCSM), Friedrich Schiller University Jena, Philosophenweg 7, 07743 Jena, Germany.

[‡]Authors contributed equally

*Correspondence to A. Traeger (anja.traeger@uni-jena.de)

List of Tables. 3
List of Figures. 3
ADDITIONAL METHODS 4
Materials. 4
Instruments. 5
Detailed Polymer Synthesis and Characterization. 6
Calculations for RAFT Polymerization. 10
Dynamic and Electrophoretic Light Scattering (DLS & ELS). 12
Cryo Transmission Electron Microscopy (cryo-TEM). 13
Titration. 14
N*/P Ratio Calculations. 15
Ethidium Bromide Quenching Assay (EBA) and Heparin Dissociation Assay (HRA). 16
Determination of Cytotoxicity. 17
Erythrocyte Aggregation and Hemolysis. 19
Polyplex Uptake *via* Flow Cytometry 20
Polyplex Uptake *via* CLSM. 21
Image Acquisition and Processing for the Calcein Release Assay *via* CLSM. 24
FURTHER RESULTS 26
Characterization of Polymers. 26
Original Cryo-TEM images 30
DLS Measurements. 32
pDNA Binding Assays. 38
Cytotoxicity Assays. 39
Microscopic Images of Treated HEK293T Cells. 40
Transfection Efficiency. 41
Polyplex Uptake. 45
Calcein Release. 48

List of Tables.

Table S1. Amount of different substances used for polymerization of block copolymers. 11
Table S2. Amount of different substances used for polymerization of (shielding) polymers. 11
Table S3. Kinetic cycle protocol for automated heparin addition by the microplate reader........ 17
Table S4. Overview of the different assembly batches of HC- and HAC-mic. 31
Table S5. Polymer concentrations in different assays.. 31
Table S6. Summary of micelle characterization at different pH-values, high concentrations..... 32
Table S7. Summary of micelle characterization at different pH values, low concentrations. 32
Table S8. MFI values of different controls in flow cytometry... 43

List of Figures.

Figure S1. Characterization of $PDMAEAm_{82}$.. 26
Figure S2. NMR results of the HC-mic in $CDCl_3$... 27
Figure S3. NMR results of the HAC-mic. 28
Figure S4. Characterization of the layer diblock copolymer. 29
Figure S5. Original cryo-TEM images of micelles. 30
Figure S6. DLS measurements of micelle stock solutions directly after formulation. 33
Figure S7. DLS measurements of (layered) micelles at different pH values.............................. 34
Figure S8. DLS measurements of (layered) polyplexes in HBG buffer. 35
Figure S9. DLS measurements of different batches of (layered) micelles. 36
Figure S10. DLS/ELS measurements of different batches of (layered) polyplexes. 37
Figure S11. Additional EBA&HRA results. 38
Figure S12. Additional cytotoxicity assays.. 39
Figure S13. Influence of (layered) polyplexes on cell morphology. 40
Figure S14. Influence of storage time on transfection efficiency. 41
Figure S15. Influence of different assembly batches on transfection efficiency. 41
Figure S16. Transfection efficiency with increased pDNA concentration in HEK293T cells. ... 42
Figure S17. Influence of concentration on transfection efficiency. 42
Figure S18. Gating strategy for pDNA transfection using examples of 24 h incubation. 44
Figure S19. Gating strategy for polyplex uptake using examples of 1 and 24 h incubation. 45
Figure S20. Quantitative analysis of polyplex uptake by CLSM. 46
Figure S21. CLSM images of polyplex uptake by HEK293T cells. 47
Figure S22. Brightfield (BF) and gray images of HEK293T cells for calcein CLSM study....... 48

ADDITIONAL METHODS

Materials.

All chemicals were used as received unless stated otherwise. The chain transfer agent 2-(Butylthiocarbonothioylthio) propanoic acid (PABTC) was prepared following a previously reported procedure.[1] Dimethylamino ethyl acrylamide (DMAEAm) was obtained from ABCR (Germany) and purified by column chromatography (silica, ethyl acetate). Sodium sulfate (Na_2SO_4) was obtained from Grüssing GmbH (Germany). Trifluoroacetic acid (TFA) was obtained from TCI (Japan). Acetic acid glacial (HOAc), sodium hydrogen carbonate ($NaHCO_3$), sodium hydroxide (NaOH) and sodium chloride (NaCl) were obtained from Fisher Scientific (U.S.). 2,2'-Azobis(2,4-dimethylvaleronitrile) (V65B) was obtained from FUJIFILM Wako Chemicals (Germany). Sodium acetate trihydrate ($NaOAc \times 3H_2O$), 4-Acryloylmorpholine (NAM), 1,3,5-trioxane and anhydrous *N*,*N*-dimethylacetamide (99.8%, DMAc) were obtained from Sigma Aldrich (U.S.). 1,4-Dioxane (>99.5%) was obtained from Carl Roth (Germany). *n*-Butyl acrylate (*n*BA), hydrogen chloride (HCl) and dimethylformamide (DMF) were obtained from Alfa Aeasar (U.S.). NAM, *n*BA and 1,4-dioxane were stored over inhibitor remover beads (for hydroquinone and monomethyl ether hydroquinone) and stored at 4 °C. Tetrahydrofuran (THF), *n*-hexane, methanol and chloroform were distilled on site.

For biological studies, Dulbecco’s modified eagle medium (DMEM), 4-(2-hydroxyethyl)-1-piperazineethanesulfonic acid) (HEPES) buffer and phosphate buffered saline (PBS) were obtained from Biowest SAS (France). Fetal calf serum (FCS) was obtained from Capricorn Scientific (Germany). PrestoBlue™ solution, YOYO-1 iodide, CellMask™ Deep Red Plasma membrane (CMDR-PM) and Penicillin-Streptomycin were obtained from Thermo Fisher Scientific (U.S.). Trypsin-EDTA-solution, Triton X-100, 0.4% trypan blue solution, Hanks’

balanced salt solution (HBSS) and calcein were obtained from Sigma Aldrich (U.S.). 1% ethidium bromide solution (EtBr) was obtained from Carl Roth (Germany). Heparin sodium salt from porcine intestinal mucosa was obtained from Alfa Aesar (U. S.). Poly(acrylic acid) (PAA, MW 2000), linear poly(ethyleneimine) (LPEI, $M_w = 25$ kg mol^{-1}) and branched PEI (BPEI, $M_w = 10$ kg mol^{-1}) were obtained from Polysciences (U.S.), CytoTox-ONE^{TM} Homogeneous Membrane Integrity Assay was obtained from Promega (U.S.). Plasmid DNA (pDNA) encoding mEGFP-N1 and pKMyc were gifts from Michael Davidson (Addgene plasmid #54767; http://n2t.net/addgene:54767; RRID: Addgene_54767) and Ian Macara (Addgene plasmid #19400; http://n2t.net/addgene:19400; RRID: Addgene_19400), respectively, and were isolated from E. Coli using a Giga plasmid kit (Quiagen, Germany).

Instruments.

Nuclear magnetic resonance (NMR) spectroscopy. ^{1}H NMR (300 MHz) and DEPT ^{13}C (75 MHz) spectra were recorded on a Bruker AC 300 MHz spectrometer at 300 K. The delay time (d1) was set at 1 s for ^{1}H NMR and 2 s for DEPT ^{13}C. Chemical shifts (δ) are reported in ppm.

Size exclusion chromatography (SEC). SEC was conducted on one of two instruments. Dimethylacetamide (DMAc)-SEC was conducted using an Agilent 1200 series instrument equipped with differential refractive index (DRI) and UV/vis (DAD) detector. The liquid chromatography system used 1 × PSS GRAM 30 Å column (300 × 0.8 mm, 10 µm particle size) and 1 × PSS GRAM 1000 Å column (300 × 0.8 mm, 10 µm particle size). The DMAc eluent contained 0.21 wt.% LiCl as additive. Samples were run at 1 mL min^{-1} at 40 °C. Analyte samples were filtered through a polytetrafluoroethylene (PTFE) membrane with 0.45 µm pore size prior to injection. Poly(methyl methacrylate) (PMMA) narrow standards were used to calibrate the SEC system. The measurements in aqueous solution for P(NAM-*b*-AA) were carried out on a Jasco

system equipped with a AS-2051 Plus autosampler, a DG-2080-53 degasser, a PU-980 pump, a RI-2031 Plus RI detector, a Jasco oven and a PSS SUPREMA guard/1000/30 Å (10 µm particle size). A mixture of 0.08 M Na_2HPO_4/0.05% NaN_3 (pH 9) was used as an eluent at a flow rate of 1 mL min^{-1} and an oven temperature of 30 °C. PEG standards (400-800,000 g mol^{-1}) were used to calibrate the system. Experimental $M_{n,SEC}$ and $Đ$ (M_w/M_n) values of synthesized polymers were determined using PSS WinGPC UniChrom GPC software.

Flow cytometry. Flow cytometry was conducted on the CytoFlex S by Beckman Coulter GmbH, Germany. For each experiment, $\geq 10^4$ cells per sample were analyzed regarding their viability, single cells and fluorescence at $\lambda_{Ex} = 488$ with a 525 nm bandpass filter (all employed stains, YOYO-1 & EGFP, were green fluorescent) in forward/sideward scatter (FSC, SSC), in FSC-Area /FSC-Height, and in FITC/SSC scatter plots, respectively.

Microplate reader. Fluorescence intensity measurements for PrestoBlue, LDH assays and absorption measurements for hemolysis and aggregation assays were performed on the Infinite M200 PRO microplate reader (Tecan, Germany) with λ_{Ex} / λ_{Em} used as indicated in the respective method sections and gain set to optimal. The combined EBA&HRA assay was conducted on the Cytation 5 multi-mode reader by BioTek, U.S.

Detailed Polymer Synthesis and Characterization.

Synthesis of P(DMAEAm)$_{82}$ was performed as described before.[2] PABTC (11.9 mg, 5.0×10^{-5} mol), DMAEAm (682.3 mg, 64.8×10^{-3} mol), 1,4-dioxane (428.5 mg, 416.0 µL), DMAc (247.8 mg, 263.6 µL), V-65B (213.1 mg of a 1 wt.% solution in 1,4-dioxane, 2.1 mg, 8.2×10^{-6} mol) and 1,3,5-trioxane (16.3 mg) as an external NMR reference were introduced to a vial equipped with a magnetic stirring bar which was sealed with a cap. The mixture was deoxygenated

by bubbling argon through the solution for 10 min. The vial was then transferred to a thermostated oil bath set at 60 °C. After a polymerization time of 4 h, the flask was cooled to room temperature (RT) and exposed to air. 2-3 droplets of the polymerization mixture were used for ^{1}H NMR and SEC analysis. Afterward, the crude polymer was precipitated three times from THF into -80 °C cold *n*-hexane. The polymer was dried under vacuum. Then, the polymer was dissolved in distilled water and lyophilized.

Synthesis of P(nBA). PABTC (230.40 mg, 9.7×10^{-4} mol), *n*BA (12389.6 mg, 9.7×10^{-2} mol), 1,4-dioxane (4164.2 mg, 4042.9 μL), V-65B (1497.0 mg of a 0.5 wt.% solution in 1,4-dioxane, 7.5 mg, 2.9×10^{-5} mol) and 1,3,5-trioxane (33.0 mg) as an external NMR reference were introduced to a vial equipped with a magnetic stirring bar which was sealed with a cap. The mixture was deoxygenated by bubbling argon through the solution for 20 min. The vial was then transferred to a thermostated oil bath set at 50 °C. After a polymerization time of 4 h, the flask was cooled to RT and exposed to air. 2-3 droplets of the polymerization mixture were used for ^{1}H NMR and SEC analysis. Afterward, the solvent was removed and the crude polymer was precipitated three times from THF into cold MeOH/ H_20 (75/25). Finally, the polymer was dried under vacuum.

Synthesis of P(nBA$_{80}$-b-DMAEAm$_{90}$). P(*n*BA)$_{80}$ (631.7 mg, 6.0×10^{-5} mol), DMAEAm (1226.2 mg, 8.6×10^{-3} mol), 1,4-dioxane (842.1 mg, 817.6 μL), V-65B (553.6 mg of a 0.5 wt.% solution in 1,4-dioxane, 2.8 mg, 1.0×10^{-5} mol) and 1,3,5-trioxane (8.7 mg) as an external NMR reference were introduced to a vial equipped with a magnetic stirring bar which was sealed with a cap. The mixture was deoxygenated by bubbling argon through the solution for 20 min. The vial was then transferred to a thermostated oil bath set at 55 °C. After a polymerization time of 70 min, the flask was cooled to RT and exposed to air. 2-3 droplets of the polymerization mixture were used

for ^{1}H NMR and SEC analysis. Afterward, the crude polymer was precipitated three times from THF into -80 °C cold *n*-hexane. Finally, the polymer was dried under vacuum.

Synthesis of P(nBA$_{86}$-b-tBA$_{43}$). P(*n*BA)$_{86}$ (1521.3 mg, 1.4×10^{-4} mol), *t*BA (868.9 mg, 6.8×10^{-3} mol), 1,4-dioxane (2491.1 mg, 2418.5 µL), V-65B (394.5 mg of a 1.0 wt.% solution in 1,4-dioxane, 4.0 mg, 1.5×10^{-5} mol) and 1,3,5-trioxane (14.3 mg) as an external NMR reference were introduced to a vial equipped with a magnetic stirring bar which was sealed with a cap. The mixture was deoxygenated by bubbling argon through the solution for 15 min. The vial was then transferred to a thermostated oil bath set at 50 °C. After a polymerization time of 5 h, the flask was cooled to RT and exposed to air. 2-3 droplets of the polymerization mixture were used for ^{1}H NMR and SEC analysis. Afterward, the crude polymer was precipitated twice from THF into cold MeOH/ H_2O (90/10). Finally, the polymer was dried under vacuum.

Synthesis of P(nBA$_{86}$-b-tBA$_{43}$-b-DMAEAm$_{88}$). P(*n*BA$_{86}$-*b*-*t*BA$_{43}$) (504.7 mg, 3.0×10^{-5} mol), DMAEAm (604.0 mg, 4.3×10^{-3} mol), 1,4-dioxane (1220 mg, 1184.5 µL), V-65B (101.9 mg of a 2.0 wt.% solution in 1,4-dioxane, 2.0 mg, 7.9×10^{-6} mol) and 1,3,5-trioxane (7.3 mg) as an external NMR reference were introduced to a vial equipped with a magnetic stirring bar which was sealed with a cap. The mixture was deoxygenated by bubbling argon through the solution for 15 min. The vial was then transferred to a thermostated oil bath set at 55 °C. After a polymerization time of 70 min, the flask was cooled to RT and exposed to air. 2-3 droplets of the polymerization mixture were used for ^{1}H NMR and SEC analysis. Afterward, the crude polymer was precipitated three times from THF into -80 °C cold *n*-hexane. The polymer was dried under vacuum.

Synthesis of P(NAM). PABTC (105.0 mg, 4.4×10^{-5} mol), NAM (4660.0 mg, 3.3×10^{-2} mol), 1,4-dioxane (8800.0 mg, 8518.9 µL), V-65B (318.6 mg of a 1.0 wt.% solution in 1,4-dioxane, 3.19 mg,

1.2×10^{-5} mol) and 1,3,5-trioxane (75.0 mg) as an external NMR reference were introduced to a vial equipped with a magnetic stirring bar which was sealed with a cap. The mixture was deoxygenated by bubbling argon through the solution for 10 min. The vial was then transferred to a thermostated oil bath set at 50 °C. After a polymerization time of 21 h, the flask was cooled to RT and exposed to air. 2-3 droplets of the polymerization mixture were used for ^{1}H NMR and SEC analysis. Afterward, the crude polymer was precipitated three times from THF into -80 °C cold *n*-hexane. The polymer was dried under vacuum.

Synthesis of P(NAM$_{74}$-b-tBA$_{42}$). P(NAM$_{74}$) (850.0 mg, 7.98×10^{-5} mol), *t*BA (511.7 mg, 3.99×10^{-3} mol), 1,4-dioxane (1800.0 mg, 1747.6 μL), V-65B (134.0 mg of a 1.0 wt.% solution in 1,4-dioxane, 1.34 mg, 5.18×10^{-6} mol) and 1,3,5-trioxane (28.0 mg) as an external NMR reference were introduced to a vial equipped with a magnetic stirring bar which was sealed with a cap. The mixture was deoxygenated by bubbling argon through the solution for 10 min. The vial was then transferred to a thermostated oil bath set at 50 °C. After a polymerization time of 6 h, the flask was cooled to RT and exposed to air. 2-3 droplets of the polymerization mixture were used for ^{1}H NMR and SEC analysis. Afterward, the crude polymer was precipitated three times from chloroform into cold *n*-hexane. Finally, the polymer was dried under vacuum.

Boc-deprotection of P(nBA$_{86}$-b-tBA$_{43}$-b-DMAEAm$_{88}$). A sample of Boc-protected polymer was introduced to a 25 mL round-bottom flask equipped with a magnetic stirring bar and TFA/deionized water (97/3, v/v) was added to reach a concentration of 147 mg mL^{-1}. A small amount of THF was added to aid the solubility. The solution was stirred for 3 h at RT and the TFA was blown off overnight using compressed air. Subsequently, the crude deprotected polymer was precipitated three times from THF into -80 °C cold hexane. Finally, the deprotected polymer was dried under vacuum.

Boc-deprotection of P(NAM$_{74}$-b-tBA$_{42}$). A sample of Boc-protected polymer was introduced to a 25 mL round-bottom flask equipped with a magnetic stirring bar and TFA/DMF (34/66, v/v) were added. The solution was stirred overnight at RT and quenched with sat. $NaHCO_3$ to reach pH 7. Then, the solution was dialyzed in water over 2 d with changing solution every 2 h for the first 8 h, and twice the following 2 d. After dialysis, the solution was concentrated under vacuum and lyophilized to yield the product.

Calculations for RAFT Polymerization.

The monomer conversion (p) was calculated from ^{1}H NMR data by comparing the integrals of vinyl peaks (5.5-5.75 ppm) against the external reference 1,3,5-trioxane (5.10 ppm) before and after polymerization. The theoretical number-average molar mass ($M_{\mathrm{n,th}}$) was calculated with Equation S1:

$$M_{\mathrm{n,th}} = \frac{[M]_0 p M_{\mathrm{M}}}{[CTA]_0} + M_{\mathrm{CTA}} \qquad (S1)$$

$[M]_0$ and $[CTA]_0$ are the initial concentrations of monomer and chain transfer agent (CTA), respectively. M_{M} and M_{CTA} are the molecular masses of the monomer and CTA, respectively.

Table S1. Amount of different substances used for polymerization of block copolymers.

Assembly code	-	HC	-	-	HAC
Composition	P(*n*BA)$_{80}$	P(*n*BA$_{80}$-*b*-DMAEAm$_{90}$)	P(*n*BA)$_{86}$	P(*n*BA$_{86}$-*b*-*t*BA$_{43}$).	P(*n*BA$_{86}$-*b*-*t*BA$_{43}$-*b*-DMAEAm$_{88}$)
Monomer	*n*BA	DMAEAm	*n*BA	*t*BA	DMAEAm
$DP_{n,target}$	100	145	100	50	140
m_{CTA} added (mg)	230.4	631.7	185.9	1521.3	504.7
n_{CTA} added (mol)	9.66×10^{-4}	5.97×10^{-5}	7.80×10^{-4}	1.36×10^{-4}	3.02×10^{-5}
$m_{monomer}$ added (mg)	12389.6	1226.2	9996.2	868.9	604.0
$n_{monomer}$ added (mol)	9.67×10^{-2}	8.63×10^{-3}	7.80×10^{-2}	6.78×10^{-3}	4.25×10^{-3}
$m_{V\text{-}65B}$ added (mg)	7.49	2.77	8.63	3.95	2.04
$n_{V\text{-}65B}$ added (mol)	2.90×10^{-5}	1.07×10^{-5}	3.34×10^{-5}	1.53×10^{-5}	7.89×10^{-6}
Dioxane added (g)	4164.2	842.1	6216.4	2491.1	1220.0
CTA/V-65B	33.3	5.6	23.3	9.0	3.9
T (°C)	50	55	50	50	55
Time (min)	240	70	300	300	65

Table S2. Amount of different substances used for polymerization of (shielding) polymers.

Assembly code	C	S	-	SA
Composition	P(DMAEAm)$_{82}$	P(NAM)$_{72}$	P(NAM)$_{74}$	P(NAM$_{74}$-*b*-*t*BA$_{42}$)
Monomer	DMAEAm	NAM	NAM	*t*BA
$DP_{n,target}$	96	75	75	50
m_{CTA} added (mg)	11.9	105.0	105.0	850.0
n_{CTA} added (mol)	4.99×10^{-5}	4.40×10^{-5}	4.40×10^{-5}	7.98×10^{-5}
$m_{monomer}$ added (mg)	682.3	4660.0	4660.0	511.7
$n_{monomer}$ added (mol)	4.80×10^{-3}	3.30×10^{-2}	3.30×10^{-2}	3.99×10^{-3}
$m_{V\text{-}65B}$ added (mg)	2.13	3.19	3.19	1.34
$n_{V\text{-}65B}$ added (mol)	8.25×10^{-6}	1.23×10^{-5}	1.23×10^{-5}	5.18×10^{-6}
Dioxane added (g)	428.5	8800.0	8800.0	1800.0
DMAc added (g)	247.8	-	-	-
CTA/V-65B	6.2	3.6	3.6	15.4
T (°C)	60	50	50	50
Time (min)	240	1260	1260	360

Dynamic and Electrophoretic Light Scattering (DLS & ELS).

The hydrodynamic diameters and ζ-potential of the nano assemblies were monitored for three different sample preparations similar to as described before[3] by DLS or ELS using a Zetasizer Nano ZS (Malvern Instruments, Germany) with a He−Ne laser operating at a wavelength of 633 nm. The sample preparations were i) pure micelle solutions as obtained after dialysis, ii) micelle solutions mixed 3+1 with shielding polymer solution or buffer at different pH values, and iii) polyplexes of micelles mixed 3+1 with shielding polymer solution or buffer as control. Regarding the pure micelle suspensions, no further sample preparation was necessary. Each sample was measured in triplicates at 25 °C with measurement duration of five times 60 s after an equilibration time of 60 s. The counts were detected at an angle of 173°. The mean particle size was approximated as the effective (z-average) diameter and the width of the distribution as the polydispersity index of the particles (PDI) obtained by the cumulants method assuming a spherical shape. The curves and data are presented in Figure S6 and Figure S9, respectively.

For the measurement of the second sample preparation, layered micelles, the samples were prepared similar to the polyplex and layering protocol, but without pDNA and at higher polymer concentrations keeping the amount of amine moieties constant within all samples. The micelle solutions were prepared by dilution with 50 mM acetate buffer, pH 5. For measurements at pH 7.4, 1 M NaOH was added to a final concentration of 20 mM. The shielding polymer solutions were prepared fourfold concentrated by diluting the polymer stock solutions in 100 mM acetate-HEPES buffer of the respective pH value (50 mM acetate + 50 mM HEPES, pH 5.0 or pH 7.4). Subsequently, the micelle suspension was slowly added to the shielding polymer solution (3+1 volume ratio) and carefully resuspended, obtaining either a molar PNAM/P*n*BA ratio of 1.0 or a carboxy to amine group (COOH/NH) ratio of 0.5. Where no layering was required, the shielding

polymer solution was replaced by acetate-HEPES buffer of the respective pH value. The samples were incubated at RT for 15 min and measured as described above but with measurement duration of three times 30 s after an equilibration time of 30 s. The samples were measured again after dilution of 1:3 with ultrapure water. Subsequently, ζ-potential of the diluted samples was measured in triplicates at 25 °C and 40 mV with measurement duration set to automatic (10-20 runs) after an equilibration time of 30 s and with a delay of 30 s between each measurement. Data are expressed as mean ± SD of two by three measurements (n = 2).

The third class of samples, the polyplexes, was measured following polyplex preparation at N*/P 30 in 75 µL HBG buffer and mixing with 25 µL shielding polymer solution or HBG buffer as described in the polyplex preparation section. The hydrodynamic diameter and ζ-potential of the samples were measured as described in the paragraph above, but this time the samples were diluted 1:8. Data are expressed as mean ± SD of two by three measurements (n = 2).

Cryo Transmission Electron Microscopy (cryo-TEM).

The samples for cryo-TEM were prepared as described for the second preparation for DLS and ELS measurements, but only in acetate-HEPES buffer of pH 5.0. For the pure HC and HAC assemblies, the stock solutions of the micelles in 50 mM acetate buffer were used. Cryo-TEM images were acquired with a 120 kV FEI Tecnai G2 20 equipped with a 4k × 4k Eagle HS CCD and an Olympus MegaView camera (1379 × 1024 pixels) for overview images. Sample preparation was performed by plunge-freezing the samples with a Vitrobot Mark IV system. 8.5 µL of the aqueous solutions were blotted (blot force -2; blotting time 1 s) on Quantifoil grids (R2/2, Quantifoil, Jena, Germany) and were vitrified in liquid ethane. The grids were rendered hydrophilic by Ar-plasma cleaning for 30 s (Diener Electronics, Germany). Prior to sample preparation, samples were stored in liquid nitrogen until transfer to the cryo holder (Gatan 626).

Transfer to the microscope was performed with a Gatan cryo stage and the temperature was maintained below -172 °C at all times after vitrification.

The size of the micelles was determined using ImageJ, version 1.52.[4] Briefly, hexagonal arrangements of seven micelles each were identified and the distance between the core of the center micelle and the core of each micelle in a corner was measured. For the estimation of the size of the micellar core, the diameter of a circle drawn around the micellar core was measured. The results are presented as mean ± SD of all measurements of the respective sample.

Titration.

Titration of the polymers was conducted using a Metrohm OMNIS integrated titration system. For a typical measurement, the polymers were dissolved at 5 mg mL^{-1} in 150 mM NaCl (in ultrapure water). In case of $PDMAEAm_{82}$, the solution was acidified with addition of 1 M HCl (pH ~ 2), the PAA solution was alkalized with addition of 1 M NaOH solution (pH ~ 11). The polymers were titrated (with dynamic flow rate adjustment) against 0.1 M NaOH solution up to a pH value of 12 or against 0.1 M HCl up to a pH value of 2, respectively.

The degree of charge (DOC) at different pH values was calculated as the amount of negatively or positively charged units per total amount of carboxy or amine groups, respectively (Equations S2-3)[5]:

$$\text{R-NH}^{+}(\text{CH}_2)_2\text{+OH}^{-}\text{+H}_2\text{O}\rightleftharpoons\text{R-N(CH}_2)_2\text{+H}_3\text{O}^{+}\text{+OH}^{-}$$

$$\text{DOC (PDMAEAm)} = \frac{[\text{R-NH}^{+}(\text{CH}_2)_2]}{[\text{R-N(CH}_2)_2]_{\text{tot}}} \cdot 100 \quad \text{(S2)}$$

$$\text{R-COO}^{-}\text{+H}^{+}\text{+2 H}_2\text{O}\rightleftharpoons\text{R-COOH+H}_3\text{O}^{+}\text{+OH}^{-}$$

$$\mathrm{DOC(PAA)} = \frac{[\mathrm{R\text{-}COO^-}]}{[\mathrm{R\text{-}COOH}]_{tot}} \cdot 100 \quad \text{(S3)}$$

Subsequently, a logistic curve was fitted to the obtained DOC values using Origin Pro, Version 2020b (OriginLab Corporation, US). The pK_a values were calculated as the pH value where the DOC was 50% (y = 50) by substitution into the respective functions of the logistic curves (Equation S4).

$$y = \frac{A_1 - A_2}{1+(x/x_0)^p} + A_2 \quad \text{(S4)}$$

Where A_1, A_2, x_0 and p are the initial value, the final value, the center and the power of the curve, respectively.

N*/P Ratio Calculations.

The N*/P ratio was defined as the ratio of the total amount of protonatable amines in polymer solution in relation to the total amount of phosphates in the pDNA solution.

The volume of polymer needed to prepare polyplexes with 15 µg mL^{-1} pDNA at different N*/P ratios was calculated as described by the following equations:

$$V_{total} \cdot P = V_{poly} \cdot N_{poly}$$

$$V_{poly} = \frac{V_{total} \cdot P}{N_{poly}}$$

$$V_{poly} = V_{total} \cdot \frac{n_{pDNA} \cdot P}{n_{poly} \cdot N}$$

$$V_{poly} = V_{total} \cdot \frac{m_{pDNA} \cdot P \cdot M_{poly}}{m_{poly} \cdot N \cdot M_{pDNA}}$$

Where V_{total}, P, V_{poly} and N_{poly} are the total required volume, the total number of phosphates of the pDNA, the required volume of polymer and the total number of active amines of the polymer, respectively.

Ethidium Bromide Quenching Assay (EBA) and Heparin Dissociation Assay (HRA).
The formation of polyplexes with pDNA was identified *via* quenching of ethidium bromide (EtBr) fluorescence by polymers interacting with pDNA as described before.[2] Briefly, 40 µg mL^{-1} pKMyc pDNA in HBG buffer (pH 7.4) were incubated with EtBr (1 µg mL^{-1}) at RT for 10 min. The polymer solutions were prepared by dilution with HBG buffer (pH 7.4) to give an N*/P ratio of 30. Subsequently, the pDNA-EtBr solution was mixed 1+1 with the different polymer solutions in black 96-well plates (Nunc, Thermo Fisher, Germany) and incubated at 37 °C for 15 min. Meanwhile, four times concentrated shielding polymer solutions were prepared to yield a molar PNAM/P*n*BA ratio of 1.0 or a carboxy to amine ratio of 0.5, respectively. They were added to the polyplex solutions in a ratio of 1+3, followed by careful resuspension and further 5 min incubation at 37 °C before measuring the fluorescence intensity at λ_{Ex} = 525 nm / λ_{Em} = 605 nm. Where no layering was desired, the same amount of HBG buffer was added instead of the shielding polymer solution. A sample containing only pDNA and EtBr was defined as maximum fluorescence (100%).

For the heparin dissociation assay, heparin was added to the formed polyplex-EtBr mixtures using the dispenser of the microplate reader to obtain the indicated concentrations (Table S3). After each addition, the plate was shaken, incubated at 37 °C for 10 min and fluorescence intensity was measured.

Table S3. Kinetic cycle protocol for automated heparin addition by the microplate reader

Kinetic cycle	Repetitions	*Addition of heparin*		*Orbital shake*	*Incubation*	*Measurement*
		V / µL	Stock Solution / U mL^{-1}			
1	2	5	100	10 s	10 min, 37 °C	λ_{Ex} = 525 nm / λ_{Em} = 605 nm
2	1	15	100	10 s	10 min, 37 °C	λ_{Ex} = 525 nm / λ_{Em} = 605 nm
3	3	5	500	10 s	10 min, 37 °C	λ_{Ex} = 525 nm / λ_{Em} = 605 nm
4	1	10	500	10 s	10 min, 37 °C	λ_{Ex} = 525 nm / λ_{Em} = 605 nm

The percentage of EtBr displaced upon polyplex formation or re-intercalating following pDNA release by heparin was calculated using Equation S5:

$$\text{rFI} / \% = \frac{F_{\text{Sample}}}{F_{\text{pDNA}}} \cdot 100 \qquad \text{(S5)}$$

Where rFI is the relative fluorescence intensity and F_{Sample}, and F_{pDNA} are the fluorescence intensities of a given sample and the EtBr intercalated into pDNA alone (in the case of the HRA with heparin), respectively. Data are expressed as mean ± SD of three independent determinations. The heparin concentration needed to release 50% of pDNA was calculated with OriginPro, Version 2020b (OriginLab Corporation, US) using a logistic function fitted to the respective single measurement points (n = 3) of each polymer (S4). The HC_{50}-values (y = 50) were calculated by substitution of the respective values into the equation.

Determination of Cytotoxicity.

For determination of cytotoxicity of the polymers, the PrestoBlue™ assay was performed with the L-929 cells based on ISO10993-5. In detail, cells were seeded at 0.1 × 10^6 cells mL^{-1} in growth medium (D10) containing 10 mM HEPES (D10H) in a 96-well plate without using the outer wells. Following incubation, the medium was changed to fresh D10H 1 h prior to treatment. The cells were treated in sextuplicates with polymers at different concentrations, ranging from 5 µg mL^{-1} to 130 µg mL^{-1} for 24 h. The medium was replaced by a 10% (v/v) PrestoBlue™ solution in fresh culture medium, prepared according to the manufacturer's instructions. Following an incubation

at 37 °C for 45 min, the fluorescence was measured at λ_{Ex} = 570 / λ_{Em} = 610 nm. Non-treated control cells on the same plate were referred to as 100% viability. Values above 70% were regarded as non-toxic. To assess the toxicity of polyplexes used for transfection, HEK293T cells were seeded at 0.2 × 10^6 cells mL^{-1} in D10H in a 24-well plate and incubated at 37 °C in a humidified 5% (v/v) CO_2 atmosphere for 24 h. 1 h prior to treatment the medium was changed to fresh D10H and the cells were treated with the polyplexes with or without layering at N*/P 30 and a final pDNA concentration of 1.5 µg mL^{-1} for 24 h prior to the PrestoBlue™ assay. The (layered) polyplexes were prepared as described in the main article with isolated pKMyc pDNA and added to the cells diluting the polyplexes 1:10 in the cell culture medium. Data are expressed as mean ± SD of at least three independent determinations

For determination of the release of lactate dehydrogenase (LDH) due to membrane disruption, the CytoTox-ONE™ assay (LDH-assay) was performed according to the manufacturer's instructions following incubation of the HEK293T cells with polyplexes with or without layering as described above in a 24-well plate for 24 h. The supernatant was transferred to a new 96-well plate as a triplicate and allowed to cool down to RT for 30 min. Subsequently, the substrate mixture including assay buffer was added and incubated at RT for 10 min. The fluorescence intensity was measured at λ_{Ex} = 560 nm / λ_{Em} = 590 nm following the addition of the stop solution. For the positive control (100% LDH release), cells were incubated with 0.2% Triton X-100 for 30 min prior to analysis. Cells incubated with only pDNA were used as negative control (0% LDH-release). The relative number of viable cells with intact membranes was calculated as follows (Equation S6):

$$\text{Viability / \%} = 100 - \frac{F_{\text{Sample}} - F_0}{F_{\text{Positive control}} - F_0} \cdot 100 \qquad \text{(S6)}$$

Where F_{sample}, F_0, and $F_{Positive\ control}$ represent the fluorescence intensity of a given sample, medium without cells, and of the Triton X-100 treated cells, respectively.

Erythrocyte Aggregation and Hemolysis.

The interaction of polymers with cellular membranes was examined by analyzing the release of hemoglobin from erythrocytes as published before.[2, 6] Blood from human donors, collected in tubes with citrate, was obtained from the Department of Transfusion Medicine of the University Hospital, Jena. The blood was centrifuged without pooling at 4,500 × g for 5 min, and the pellet was washed three times with cold phosphate buffered saline (PBS, pH 7.4). Following a 10-fold dilution with PBS (either pH 7.4 or pH 6.0), 500 µL aliquots of erythrocyte suspension were mixed 1+1 with the (layered) polymer solutions. These were prepared as described in the main article and diluted 1:5 with PBS pH 7.4 or pH 6.0. The erythrocyte-polymer suspensions were incubated at 37 °C for 60 min. After centrifugation at 2,400 × g for 5 min, the supernatant was transferred to a clear flat bottomed 96-well plate (VWR, Germany) and the hemoglobin release was determined as the hemoglobin absorption at $\lambda = 544$ nm. Absorption at $\lambda = 630$ nm was used as reference. Complete hemolysis (100%) was achieved using 1% Triton X-100 as positive control. Pure PBS was used as negative control (0% hemolysis). The hemolytic activity of the polycations was calculated as follows (Equation S7):

$$\text{Hemolysis / \%} = \frac{(A_{Sample} - A_{Negative\ control})}{(A_{Positive\ control} - A_{Negative\ control})} \cdot 100 \qquad \text{(S7)}$$

Where A_{Sample}, $A_{Negative\ control}$ and $A_{Positive\ control}$ are the absorption values of a given sample, the PBS treatment and the Triton X-100 treatment, respectively. A value less than 2% hemolysis rate was classified as non-hemolytic, 2 to 5% as slightly hemolytic and values > 5% as hemolytic.

To determine the cell aggregation, erythrocytes were isolated as described above. Subsequently, 100 µL of the erythrocyte-polymer suspension were transferred to a clear flat bottomed 96-well plate (VWR, Germany). The cells were incubated at 37 °C for 2 h, and the absorbance was measured at λ = 645 nm. Cells treated with PBS served as negative control and cells treated with 50 µg mL^{-1} 10 kDa BPEI were used as positive control. Aggregation potential of the polymers was calculated as follows (Equation S8):

$$\text{Aggregation} = \frac{A_{Negative\ control}}{A_{Sample}} \qquad \text{(S8)}$$

Where A_{Sample} and $A_{\text{Negative control}}$ are the absorption values of a given sample and the PBS treatment, respectively. Experiments were run in technical triplicates and were performed with blood from three different blood donors.

Polyplex Uptake *via* Flow Cytometry.

To study the uptake of polymers over time in HEK293T cells, the cells were seeded at 0.2 × 10^6 cells mL^{-1} in D10H in 24-well plates, followed by incubation at 37 °C in a humidified 5% (v/v) CO_2 atmosphere for 24 h and medium change to fresh D10H 1 h prior to treatment. The cells were treated with polyplexes with or without layering at N*/P 30 and a final pDNA concentration of 1.5 µg mL^{-1} for indicated time periods. The polyplexes were prepared as described above after labelling 1 µg pKMyc pDNA with 0.027 nmol YOYO-1 iodide. Subsequently, the polymer-pDNA-solutions were added to the cells, diluting the polyplexes 1:10 in cell culture medium. Following incubation, the HEK293T cells were harvested by collecting the supernatant in a separate 24-well plate, trypsinization and resuspension in the respective supernatant again. Trypan blue solution (0.4%) was added to half of the cell suspension to a final concentration of 0.04% to quench fluorescence of polyplexes outside the cells. The remaining cell suspension was diluted

1:2 with D20 and further incubated as described in the transfection section. Cells were analyzed *via* flow cytometry as described in the instrumentation section. Viable cells showing YOYO-1 signal higher than the control cells, which were incubated with YOYO-1-pDNA only, were gated as % of cells that have taken up pDNA and the rMFI of all viable cells was calculated in relation to the control cells (Figure S19). MFI values of control cells can be found in Table S8. The experiments were performed at least three times and data are expressed as mean ± SD.

Polyplex Uptake *via* CLSM.

To study the uptake of polymers *via* CLSM, HEK293T cells were seeded at 0.2 × 10^6 cells mL^{-1} in D10H in 8-well slides (ibidi, Germany), followed by incubation at 37 °C in a humidified 5% (v/v) CO_2 atmosphere for 24 h and medium change to FC-buffer (Hanks' Balanced Salt Solution, supplemented with 2% FCS and 20 mM HEPES) containing 5 µg mL^{-1} CellMask^{TM} Deep Red Plasma membrane (CMDR-PM) stain. Following incubation for 8 min, the medium was again changed to fresh D10H and incubated for 15 min prior to treatment. The cells were treated with (layered) polyplexes with or without layering at N*/P 30 and a final pDNA concentration of 1.5 µg mL^{-1}. The polyplexes were prepared as described above after labelling 1 µg pKMyc pDNA with 0.027 nmol YOYO-1 iodide. Subsequently, the polymer-pDNA-solutions were added to the cells, diluting the polyplexes 1:10 in cell culture medium. Following incubation for 1 h, the cells were incubated with with 8 µM Hoechst 33342 and 5 µg mL^{-1} CMDR-PM for 10 min. Subsequently, the medium was changed to fresh warm D20. Just before imaging of each well, trypan blue solution (0.4%) was added to a final concentration of 0.04% to quench YOYO-1 fluorescence outside the cells.

To image the intracellular distribution pattern of the polyplexes in living cells, live cell imaging was performed using a LSM880, Elyra PS.1 system (Zeiss, Germany) applying the argon laser for

excitation at 488 nm (2%), 405 nm (0.2%) and 633 nm (2%), emission filters for 410-468 nm (Hoechst), 508-553 nm (YOYO-1) and 666-755 nm (CMDR-PM) with a gain of 800, 550 and 650, respectively, and a pinhole of 68 µm. To avoid cross talk between the different channels, Hoechst 33342, YOYO-1 and CMDR-PM were imaged simultaneously in different tracks. For fast imaging, the tracks were switched in every line of the image. For magnification, a 63 × 1.4 NA plan apochromat oil objective was applied. Images were acquired using the ZEN software, version 2.3 SP1 (Zeiss, Germany). The experiments were performed three times acquiring at least 5 images per sample each time. These images consisted of three images as a z-stack with 1-2 µm between the slices. All images were processed in batch mode using the image analysis wizard of the ZEN software, version 3.1 (Zeiss, Germany) to quantify the number of polyplexes per cell nucleus and the amount of free polyplexes (YOYO-1 signal not colocalized with CMDR-PM). For the depiction of polyplex uptake, representative images of all samples were processed using ImageJ, version 1.52[4] as follows: First, the three slices of the z-stack images were combined using the maximum intensity projection method. Subsequently, the background of the Hoechst 33342 channel was corrected using the rolling ball background subtraction tool applying a sliding paraboloid with a radius of 215 pixels without previous image smoothing. The background of the YOYO-1 channel was corrected by subtraction of a mean fluorescence measured previously in spots without cells (value: 2500). The contrast of all channels was enhanced automatically with a normalization of 0.2% (YOYO-1) or 0.3% (Hoechst, CMDR-PM) saturation. For the overlay images, either all three or the YOYO-1 and the CMDR-PM channels were merged.

Regarding the quantification of polyplexes out/inside organelles, the following settings were used to extract three different feature classes, nuclei, polyplexes and organelles (with the subclass: colocalized polyplexes). Regarding the nuclei, the Hoechst channel images were smoothed (size =

3) followed by segmentation using global thresholding (10,000-65,535) with a tolerance of 3% and watershed separation (count = 25). Objects larger than 20,000,000 µm^2 were counted as nuclei. For the polyplexes, the YOYO-1 channel images were smoothed (size = 3) followed by background subtraction (radius of rolling ball = 30 µm), segmentation using global thresholding (5,500-65,535) with a tolerance of 3% and watershed separation (count = 3). Objects larger than 250 µm^2 were counted as polyplexes. Regarding the organelles, the CMDR-PM channel images were smoothed (size = 3) followed by segmentation using global thresholding (4,000-65,535) with a tolerance of 3% and dilation (count = 3). The number of polyplexes within organelles was determined as a subclass within objects designated as organelles previously using the same settings as for the polyplexes class. All steps were repeated with the same settings for all images of all samples in batch mode. The relative amount of free polyplexes was calculated using Equation S9:

$$\text{Free polyplexes / \%} = \frac{(N_{Polyplexes} - N_{Colocalized\ polyplexes})}{(N_{Polyplexes})} \cdot 100 \qquad \text{(S9)}$$

Where $N_{Polyplexes}$, and $N_{Colocalized\ polyplexes}$ are the total counts of the respective feature classes of one repetition.

The number of polyplexes per cell was calculated as follows (Equation S10):

$$\text{Polyplexes per cell} = \frac{(N_{Polyplexes})}{(N_{Nuclei})} \qquad \text{(S10)}$$

Where $N_{Polyplexes}$, and N_{Nuclei} are the total counts of the respective feature classes of one repetition. In case of the nuclei, the values were divided by three due to the acquisition of z-stack images with three slices.

Image Acquisition and Processing for the Calcein Release Assay *via* CLSM.

To image the intracellular distribution pattern of calcein in living cells, live cell imaging was performed using a LSM880, Elyra PS.1 system (Zeiss, Germany) applying the argon laser for excitation at 488 nm (1%) and 405 nm (0.5%), emission filters for 410-469 nm (Hoechst) and 490-544 nm (Calcein) with a gain of 800 and a pinhole of 27 µm, respectively. To avoid cross talk between the different channels, Hoechst 33342 and calcein were imaged simultaneously in different tracks. For fast imaging, the tracks were switched in every line of the image. For magnification, a 40 × 1.4 NA plan apochromat oil objective was applied. Images were acquired using the ZEN software, version 2.3 SP1 (Zeiss, Germany). The experiments were performed three times acquiring at least three images per sample each time. All images were processed in batch mode using ImageJ, version 1.52[4] with different macros for depiction and quantification of calcein release, respectively. For the depiction of calcein release, representative images of all samples were processed as follows: First, the background of the Hoechst 33342 channel was corrected using the rolling ball background subtraction tool applying a sliding paraboloid with a radius of 297 pixels without previous image smoothing. The contrast of both channels was enhanced automatically with a normalization of 0.01% saturation. For the overlay image, both channels were merged.

Regarding the quantification of calcein release, the images of the respective channels first had to be optimized regarding specific features (Hoechst – nuclei, calcein – extensive intracellular fluorescence). Therefore, the single channel images were made binary following special processing with the rolling ball background subtraction tool, automatic contrast enhancement and the setting of an automatic threshold. In case of the calcein channel, the “minimum” convolution filter was applied additionally before the contrast enhancement. The binary images were modified

to eliminate small holes inside the feature areas. Subsequently, the processed images of both channels were combined using the “AND” combination mode, leaving only the nuclei with coincident calcein staining, representing cells with calcein release. These were then counted *via* “Analyze Particles” setting the threshold for the size to 30 square pixels/unit. The same step was repeated with the Hoechst channel images to determine the number of nuclei per image. All steps were repeated with the same settings for all images of all samples in batch mode. A minimum of 170 cells were analyzed per sample and repetition. Finally, the proportion of cells showing calcein release was calculated as described in the main article.

FURTHER RESULTS

Characterization of Polymers.

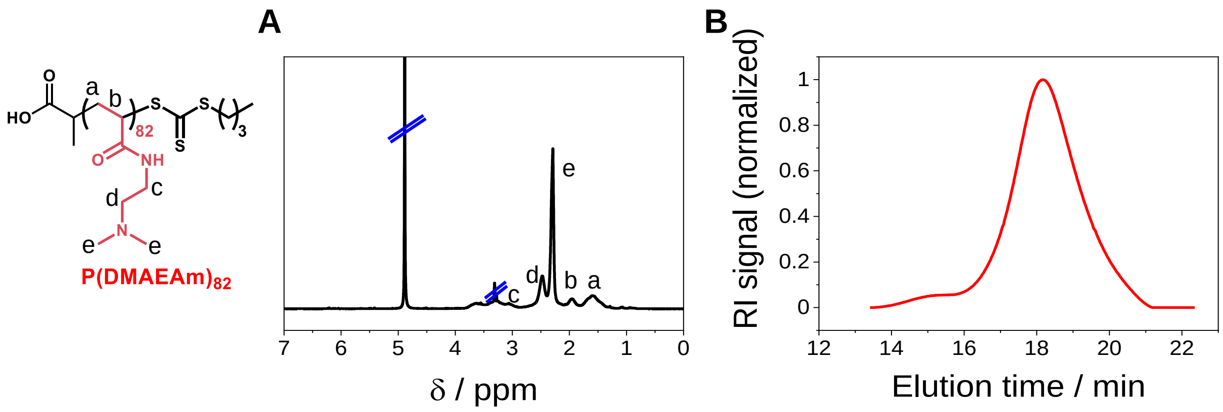

Figure S1. Characterization of PDMAEAm82.

(A) ^{1}H NMR spectrum in CD_3OD; **(B)** (DMAc + 0.21wt.% LiCl) SEC trace – PMMA calibration; $M_{n,SEC}$ =14300 g mol^{-1}, $Đ$ = 1.67.

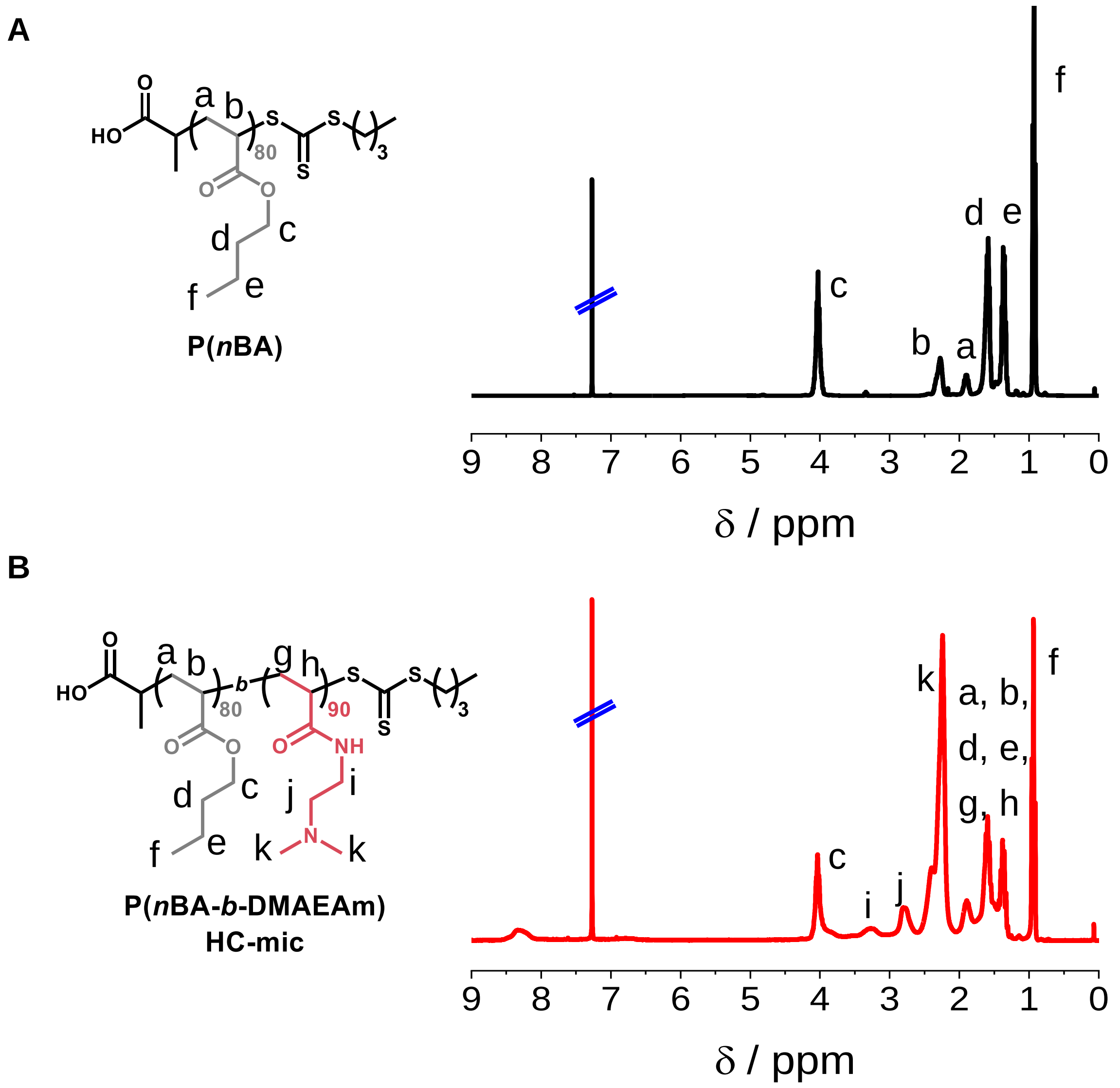

Figure S2. NMR results of the HC-mic in $CDCl_3$.

(A) P(*n*BA$_{80}$) and **(B)** P(*n*BA$_{80}$-*b*-DMAEAm$_{90}$).

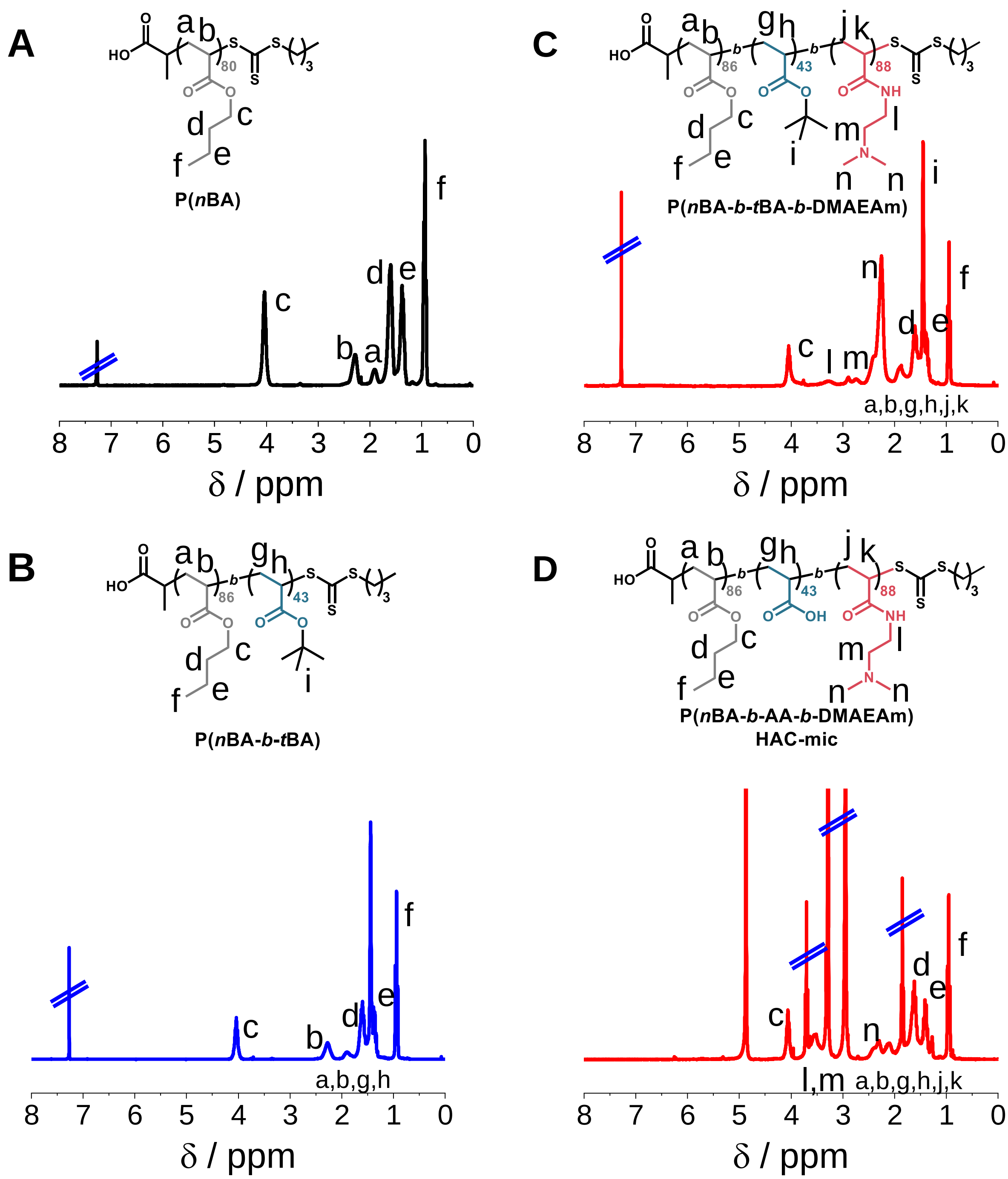

Figure S3. NMR results of the HAC-mic.

(A) P(nBA$_{86}$), **(B)** P(nBA$_{86}$-b-tBA$_{43}$), **(C)** P(nBA$_{86}$-b-tBA$_{43}$-b-DMAEAm$_{88}$) in $CDCl_3$ and **(D)** P(nBA$_{86}$-b-AA$_{43}$-b-DMAEAm$_{88}$) in CD_3OD.

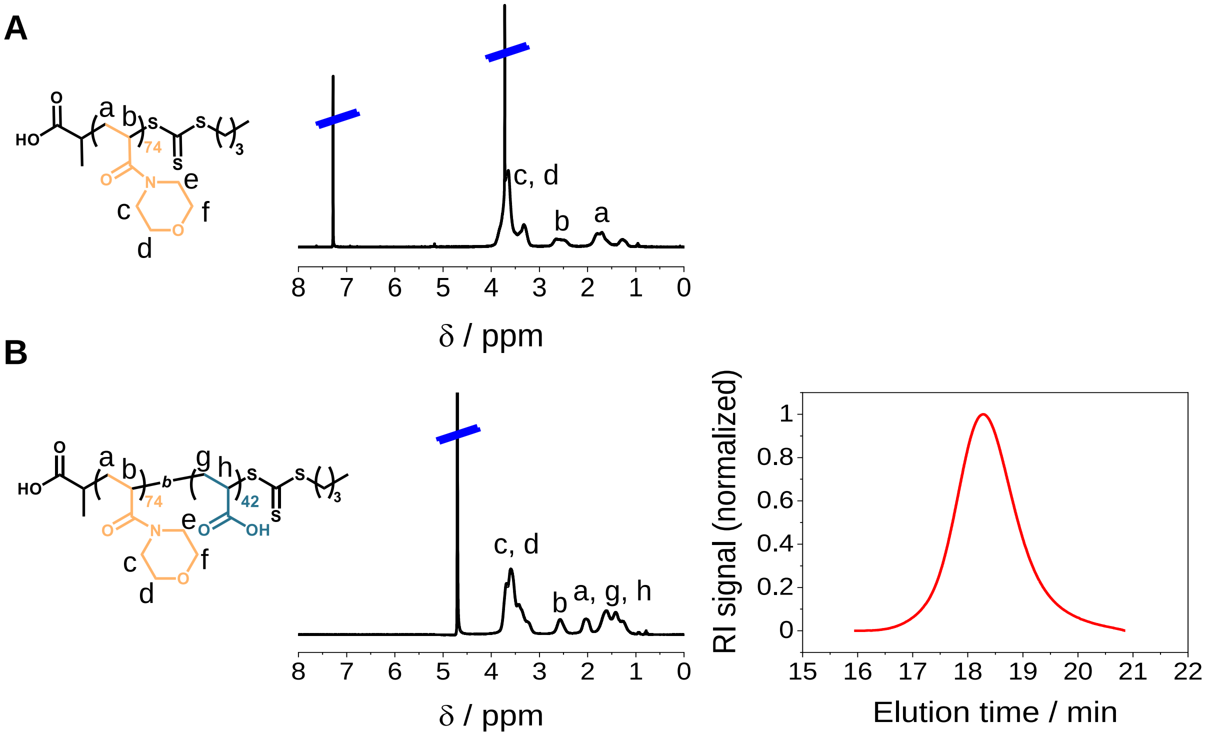

Figure S4. Characterization of the layer diblock copolymer.

(A) ^{1}H NMR of P(NAM_{74}) in $CDCl_3$, **(B)** ^{1}H NMR in D_2O and aqueous (0.08 M Na_2HPO_4 + 0.05% NaN_3) SEC trace – PEG calibration; $M_{n,SEC}$ = 17700 g mol^{-1}, $Đ$ = 1.26 of P(NAM_{74}-*b*-AA_{42}).

Original Cryo-TEM images

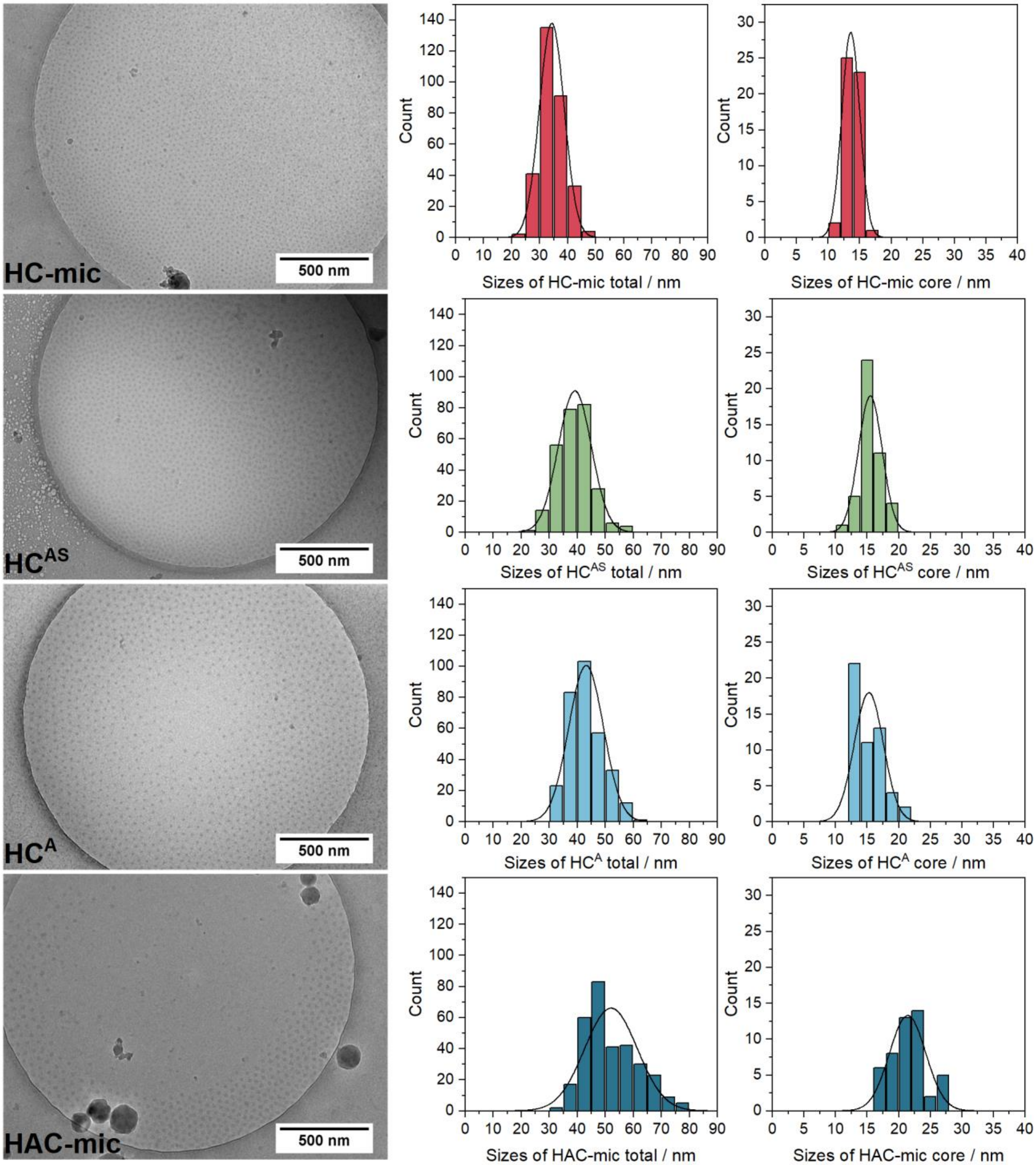

Figure S5. Original cryo-TEM images of micelles.

The samples of HC^{A} and HC^{AS} were prepared in acetate-HEPES buffer of pH 5.0 at a COOH/NH ratio of 0.5 with a final concentration of 2.7 mg mL^{-1} for the HC-mic. For the naked HC and HAC assemblies, the stock solutions of the micelles in 50 mM acetate buffer, pH 5 were used with

concentrations of 1.9 and 1.3 mg mL^{-1}, respectively. The histograms depict the size distributions of the total micelles or the micelle core within all analyzed images using ImageJ as described above. On an average, 50 micelles (*i.e.*, 300 lines, 50 circles) were measured per sample in different images.

Table S4. Overview of the different assembly batches of HC- and HAC-mic.

Polymer	Assembly	Dissolved in	Dialyzed against	Final concentration µg mL^{-1}
HC	1[a]	THF	Acetate, pH 5.9	1860
	2	THF/MeOH	Acetate, pH 5.9	1620
	3	THF/MeOH	Acetate, pH 5.0	3580
HAC	1	THF/MeOH	Acetate, pH 5.0	1740
	2	THF/MeOH	Acetate, pH 5.0	943
	3	THF/MeOH	Acetate, pH 5.0	1130
	3.2	THF/MeOH	Acetate, pH 5.0	1330

[a] Block copolymer was not treated with 0.5 eq. HCl.

Table S5. Polymer concentrations in different assays.

	DLS, pH	ELS, pH	EBA/HRA, DLS pDNA	Cell-based assays, ELS pDNA
	µg mL^{-1}	µg mL^{-1}	µg mL^{-1}	µg mL^{-1}
	-	-	N*/P 30	N*/P 30
pDNA	-	-	15	1.5
LPEI	-	-	59	5.9
PDMAEAm	-	-	229	22.9
HC-mic	845.4	281.8	357.1	35.7
HC^{S}	+ 377.7	+ 125.9	+ 159.5	+ 16.0
HC^{AS}	+ 533.4	+ 177.8	+ 225.3	+ 22.5
HC^{A}	+ 116.7	+ 38.9	+ 49.3	+ 4.93
HAC-mic	997.5	332.5	421.4	42.1

DLS Measurements.

Table S6. Summary of micelle characterization at different pH-values, high concentrations.

Code	Concentration	pH-Value	z-Average [a]	PDI [a]	Main peak [a]	Area of main peak [a]
	µg mL^{-1}		nm		nm	%
ABC-mic	998	5.0	66.4 ± 2.3	0.16 ± 0.07	66.4 ± 2.2	98 ± 5
	998	7.4	>> 1000	0.42 ± 0.26	>> 1000	100 ± 0
AC-mic	845	5.0	58.4 ± 0.9	0.17 ± 0.01	65.1 ± 5.4	98 ± 3
	845	7.4	53.0 ± 1.0	0.20 ± 0.02	51.9 ± 3.3	89 ± 6
N-AC	+378	5.0	58.1 ± 1.1	0.15 ± 0.01	64.4 ± 5.2	100 ± 0
	+378	7.4	51.5 ± 0.4	0.19 ± 0.02	52.9 ± 1.7	96 ± 4
NB-AC	+534	5.0	54.7 ± 1.2	0.13 ± 0.01	59.0 ± 3.3	99 ± 2
	+534	7.4	46.2 ± 1.2	0.20 ± 0.05	48.5 ± 2.3	94 ± 4
B-AC	+117	5.0	53.4 ± 6.5	0.20 ± 0.05	52.5 ± 6.6	93 ± 8
	+117	7.4	>> 1000	0.42 ± 0.26	>> 1000	100 ± 0

[a] Determined *via* DLS (concentrations see Table S5).
[b] Determined *via* ELS.

Table S7. Summary of micelle characterization at different pH values, low concentrations.

Code	Concentration	pH-Value	z-Average [a]	PDI [a]	Main peak [a]	Area of main peak [a]	Zeta potential [b]
	µg mL^{-1}		nm		nm	%	mV
HAC-mic	333	5.0	70.3 ± 0.5	0.10 ± 0.02	77.8 ± 1.4	100 ± 0	26 ± 5
HC-mic	219	5.0	63.3 ± 0.7	0.20 ± 0.01	68.5 ± 1.6	97 ± 1	25 ± 1
HC^{S}	+126	5.0	63.6 ± 0.9	0.20 ± 0.01	72.2 ± 3.7	98 ± 1	25 ± 2
HC^{AS}	+178	5.0	57.8 ± 0.3	0.15 ± 0.03	65.2 ± 1.5	99 ± 1	22 ± 2
HC^{A}	+39	5.0	57.2 ± 2.4	0.21 ± 0.03	64.4 ± 2.6	97 ± 2	25 ± 2
HAC-mic	333	7.4	>> 1000	0.79 ± 0.28	323 ± 505	33 ± 52	1 ± 1
HC-mic	219	7.4	55.8 ± 1.7	0.22 ± 0.03	61.4 ± 3.5	95 ± 2	16 ± 2
HC^{S}	+126	7.4	56.3 ± 3.5	0.22 ± 0.06	63.1 ± 3.7	96 ± 3	17 ± 2
HC^{AS}	+178	7.4	47.8 ± 1.4	0.26 ± 0.02	50.5 ± 1.2	92 ± 3	-2 ± 0
HC^{A}	+39	7.4	>> 1000	0.56 ± 0.34	63.1 ± 96.8	27 ± 41	9 ± 4

[a] Determined *via* DLS (concentrations see Table S5).
[b] Determined *via* ELS.

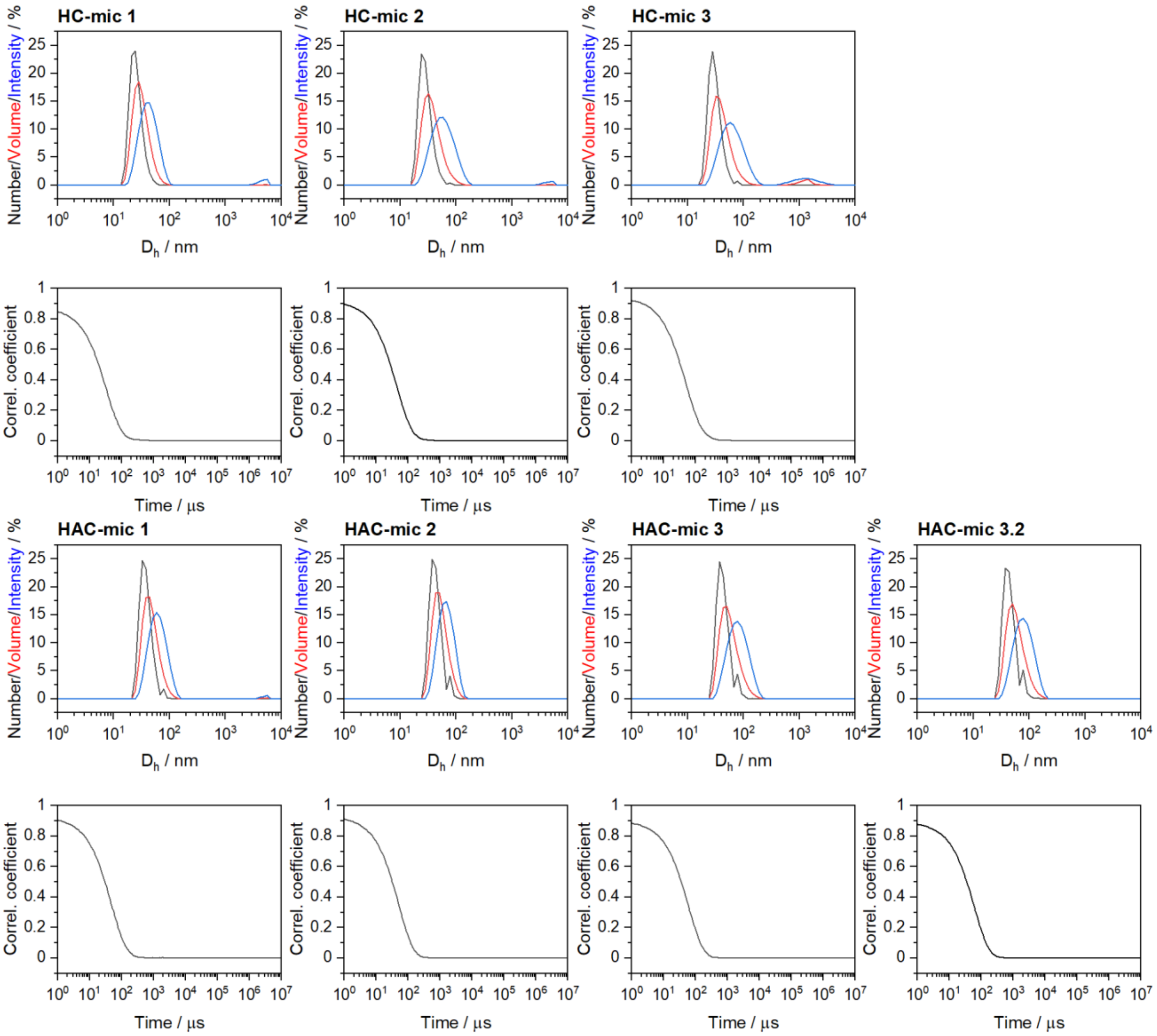

Figure S6. DLS measurements of micelle stock solutions directly after formulation.

Depiction of exemplary intensity, number, and volume weighted plots and exponential decay correlation coefficients of single measurements. Numbers indicate different formulation batches. Concentrations of the solutions were as indicated in Table S4.

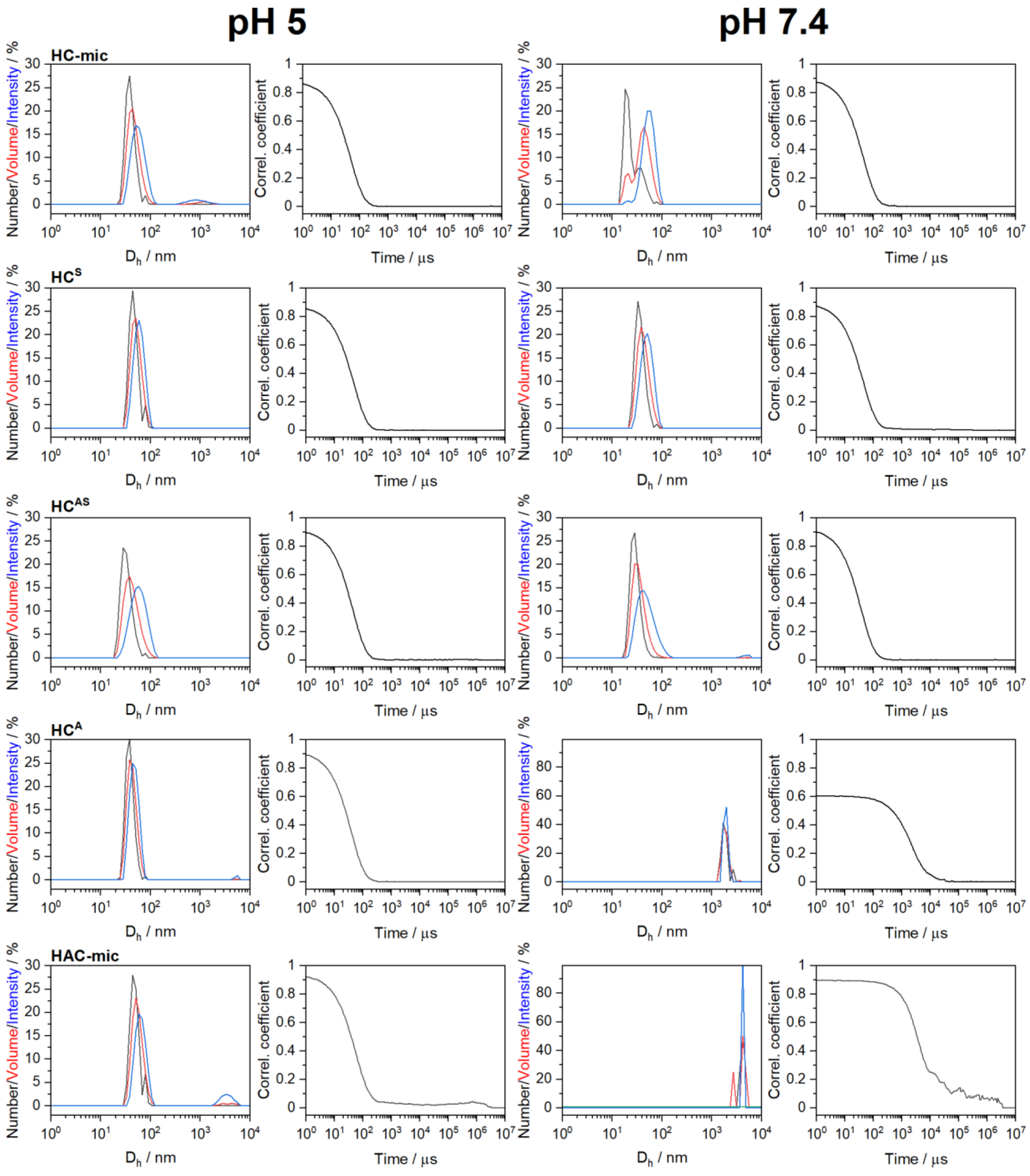

Figure S7. DLS measurements of (layered) micelles at different pH values.

Depiction of exemplary intensity, number, and volume weighted plots and exponential decay correlation coefficients of single measurements. Concentrations of the solutions were as indicated in Table S6.

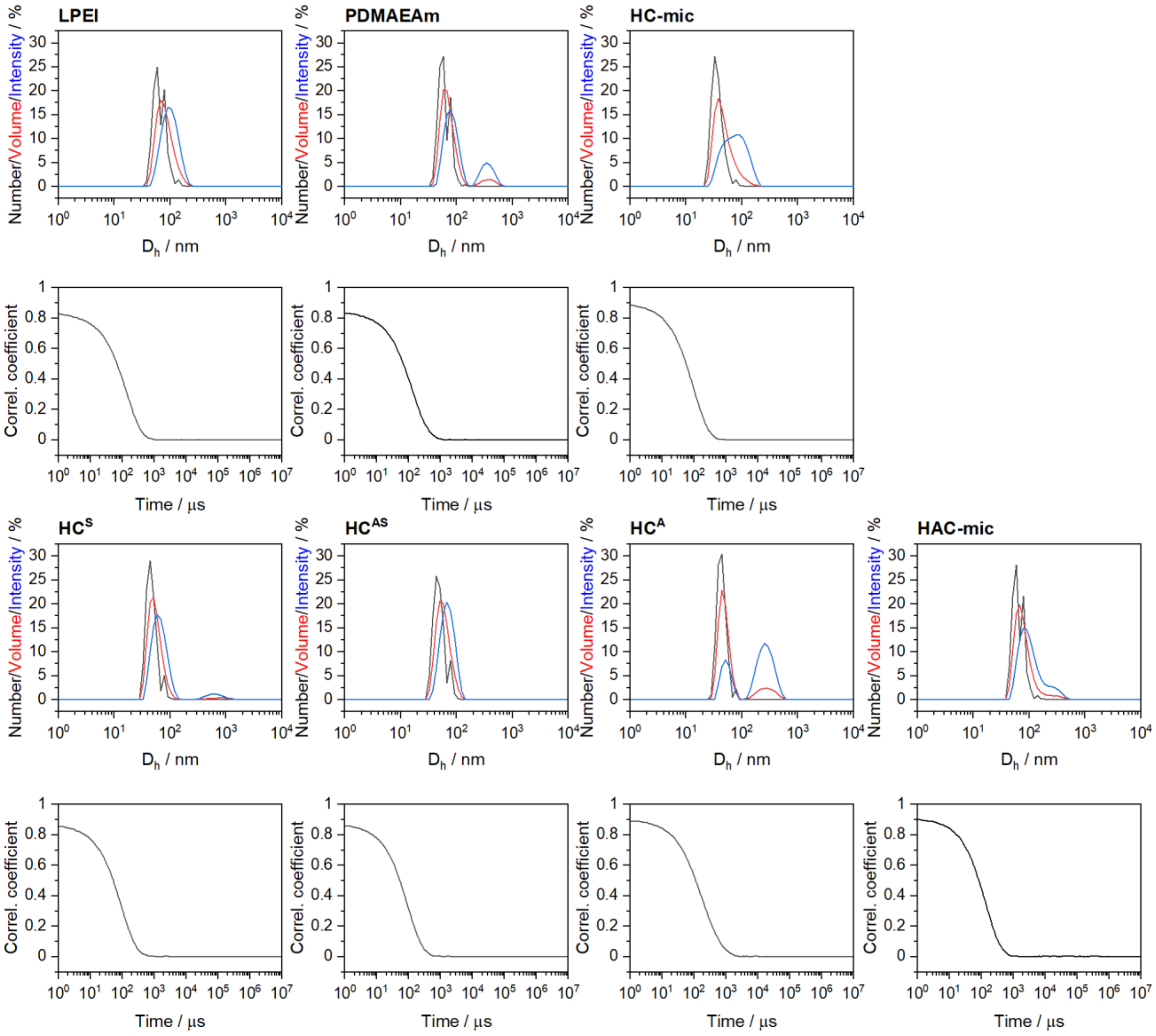

Figure S8. DLS measurements of (layered) polyplexes in HBG buffer.

Depiction of exemplary intensity, number, and volume weighted plots and exponential decay correlation coefficients of single measurements. Concentrations of the solutions were as indicated in Table S5.

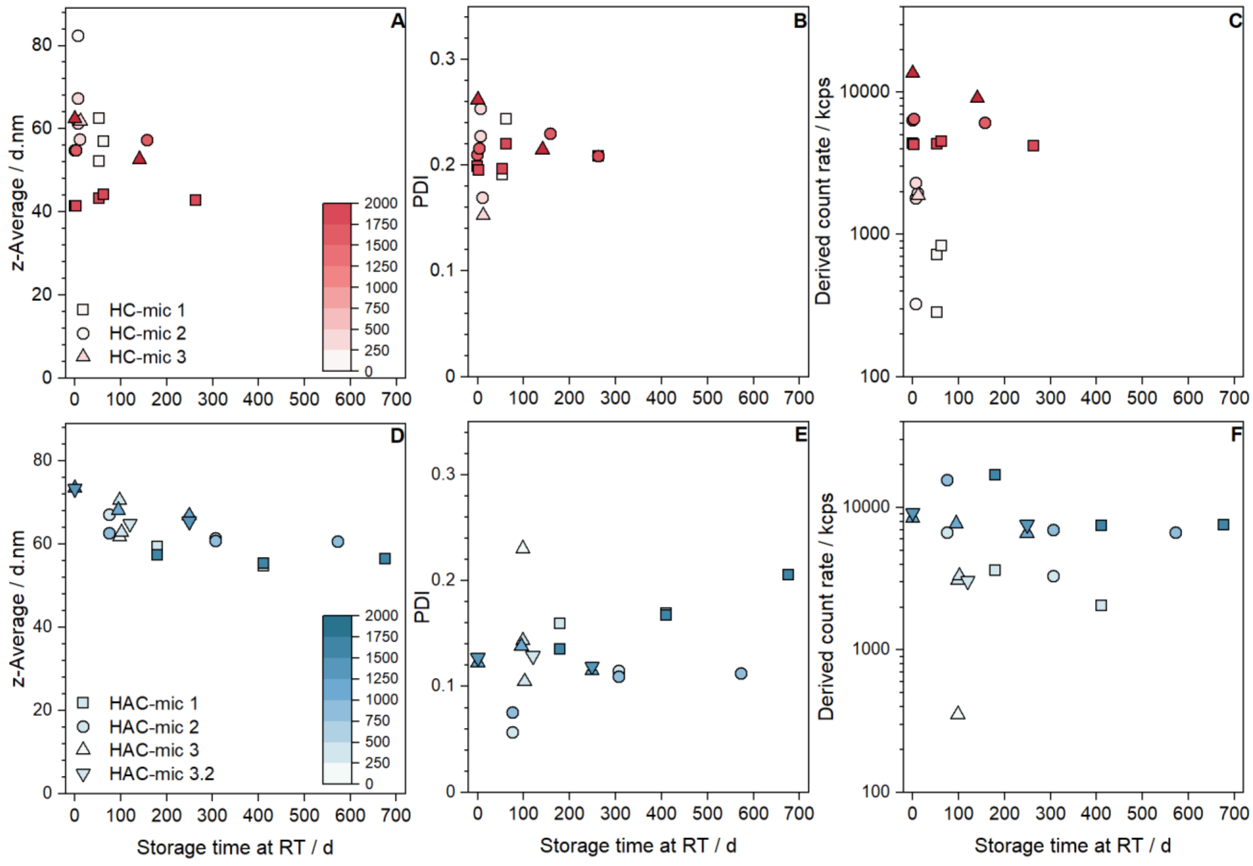

Figure S9. DLS measurements of different batches of (layered) micelles.

HC- (A-C) or HAC-micelles (D-F) were measured at different concentrations following different storage times. Different symbols represent different assembly batches (Table S4) of the respective polymer. Color code indicates polymer concentration in µg mL^{-1}.

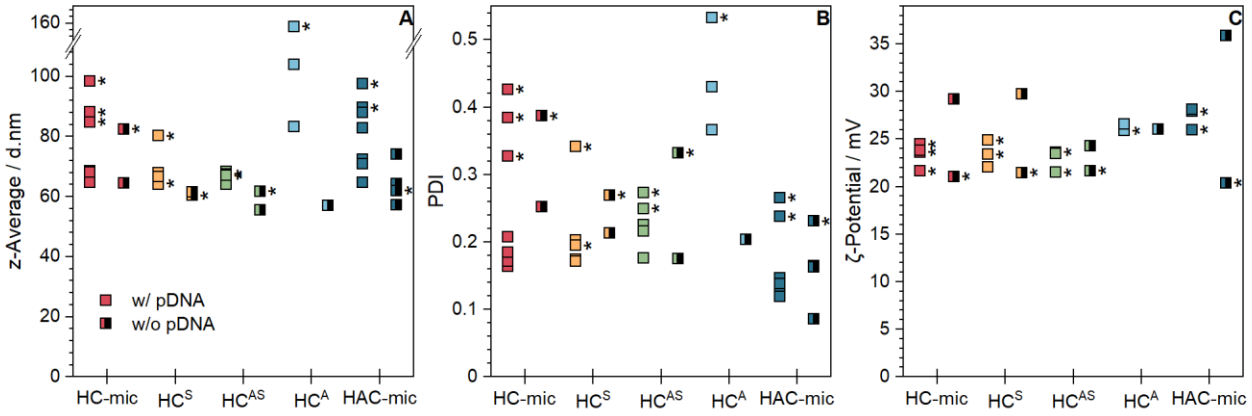

Figure S10. DLS/ELS measurements of different batches of (layered) polyplexes.

DLS/ELS measurements of (layered) polyplexes vs. (layered) micelles. Polyplexes were formed with 15 µg mL^{-1} pDNA at N*/P 30 (Table S5). Dots of the same color represent different assembly batches of the respective polymer (Table S4). Stars next to dots indicate dilution of polyplexes in HBG or water to concentrations below 100 µg mL^{-1}.

pDNA Binding Assays.

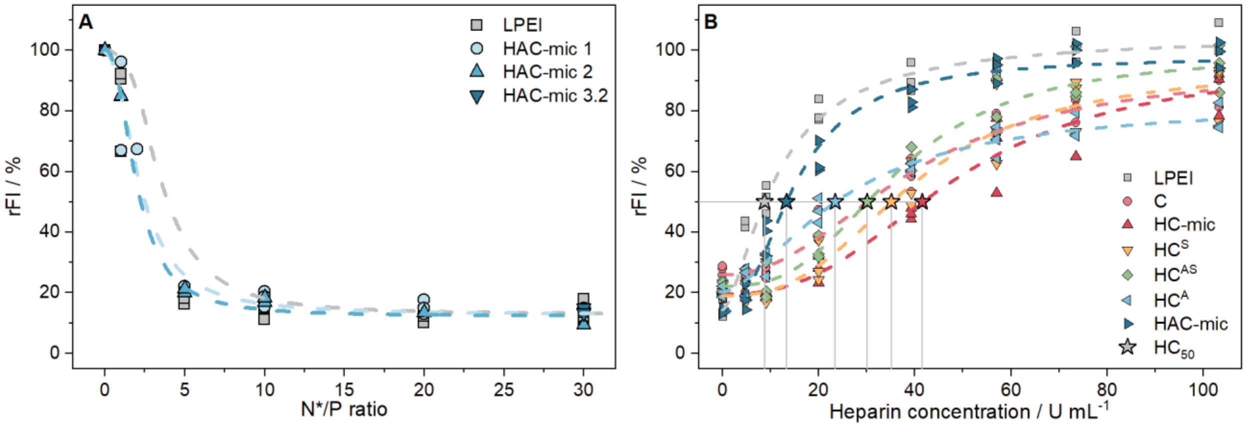

Figure S11. Additional EBA&HRA results.

(A) EBA of polyplexes of pDNA and different batches of HAC-mic (Table S4) at different N*/P ratios in HBG buffer ($n \geq 2$). (B) HRA of (layered) polyplexes at N*/P 30 ($n = 3$). Stars indicate heparin concentration needed to release 50% of pDNA (HC_{50}) as presented in the main article.

Cytotoxicity Assays.

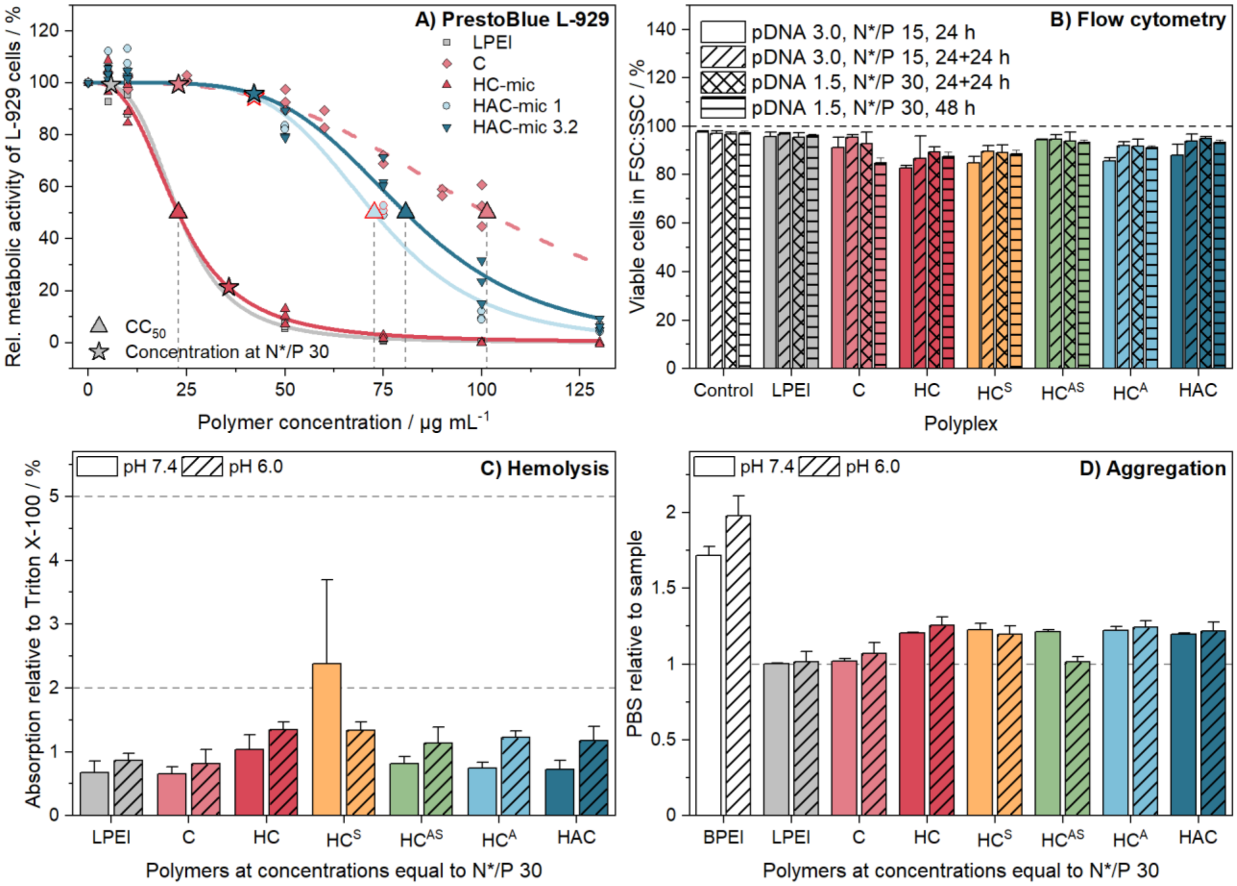

Figure S12. Additional cytotoxicity assays.

(A) Metabolic activity using the PrestoBlue™ assay following incubation of cells with polymers at indicated concentrations for 24 h. Dots represent values of single repetitions and lines represent logistic functions fitted to the single measurements (n = 3). Stars indicate concentration and viability of N*/P 30, and triangles indicate the critical concentration corresponding to 50% viable cells (CC_{50}). The curve of C was also shown in the SI of Richter *et al.* 2020.[2] (B) Hemolysis as the amount of released hemoglobin calculated relative to 1% Triton X-100 as positive control (100% hemolysis). Human erythrocytes were washed and incubated with polymers at indicated concentrations in PBS of different pH values without FCS. Values represent mean ± SD (n = 3). and are classified as slightly hemolytic between 2% and 5%, and as non- or hemolytic if lower or higher than 2% or 5%, respectively. (C) Aggregation of indicated polymers was measured as light absorption by erythrocytes. Erythrocytes were washed and incubated as described in (B). 10 kDa

BPEI was used as positive control. Values are calculated as the negative control (PBS value) relative to the sample value and represent mean ± SD (n = 3).

Microscopic Images of Treated HEK293T Cells.

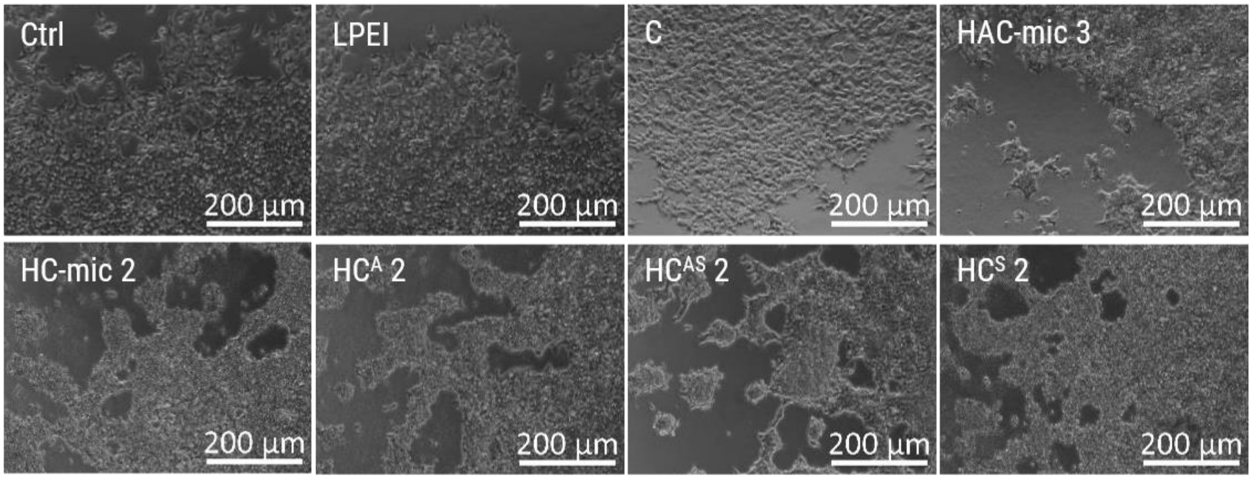

Figure S13. Influence of (layered) polyplexes on cell morphology.

Cells were incubated with (layered) polyplexes of polymers and pDNA at N*/P 30 for 24 h. Images were acquired *via* light microscopy.

Transfection Efficiency.

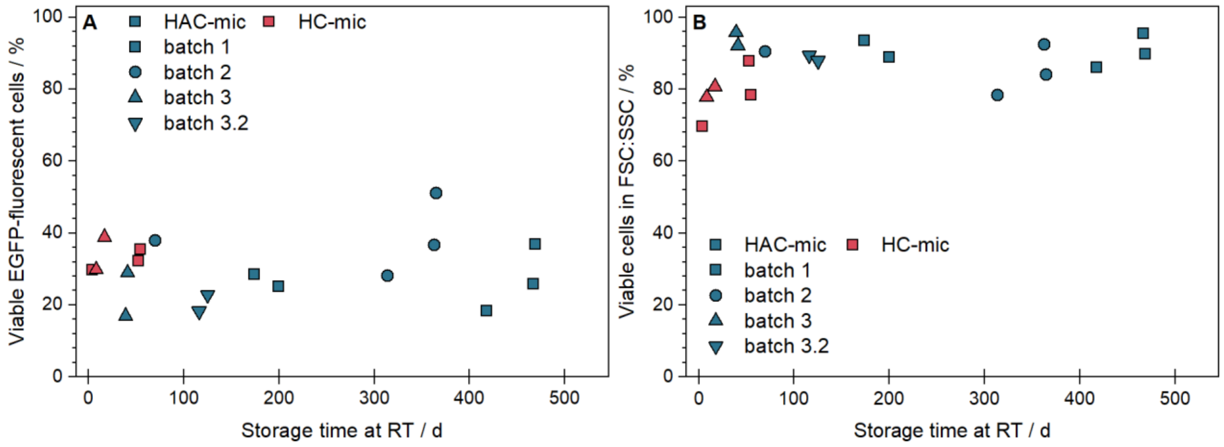

Figure S14. Influence of storage time on transfection efficiency.

Storage time dependent transfection efficiency in HEK293T cells following incubation with polyplexes of 1.5 µg mL^{-1} pDNA and micelles at N*/P 30 for 24 h. The micelle assemblies were stored at RT for indicated time periods. Values represent (A) viable EGFP fluorescent cells or (B) viable cells in FSC/SSC scatter plot of single measurements.

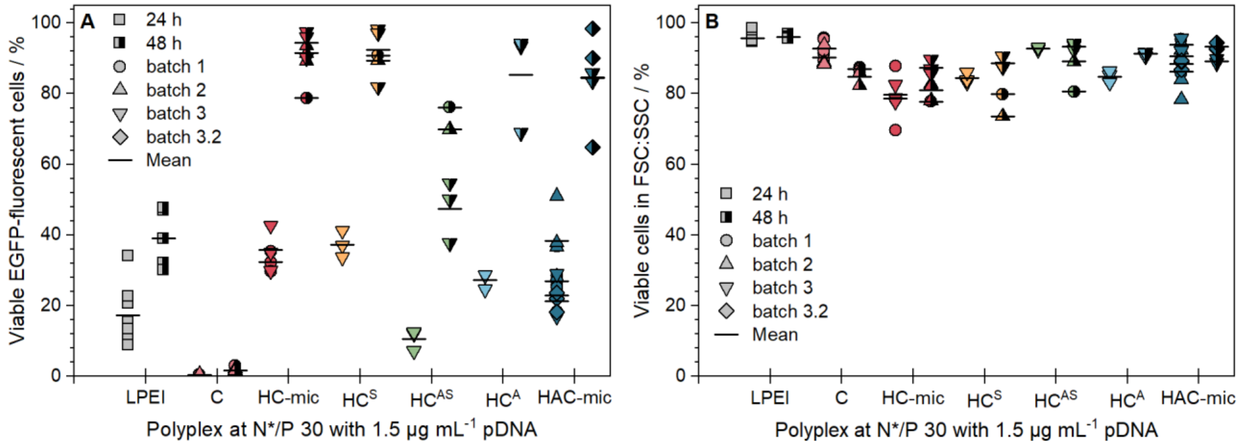

Figure S15. Influence of different assembly batches on transfection efficiency.

Batch dependent transfection efficiency in HEK293T cells following incubation with (layered) polyplexes of 1.5 µg mL^{-1} pDNA and polymers at N*/P 30 for 24 or 48 h. Values represent (A) viable EGFP fluorescent cells or (B) viable cells in FSC/SSC scatter plot of single measurements. Lines indicate the mean of one batch.

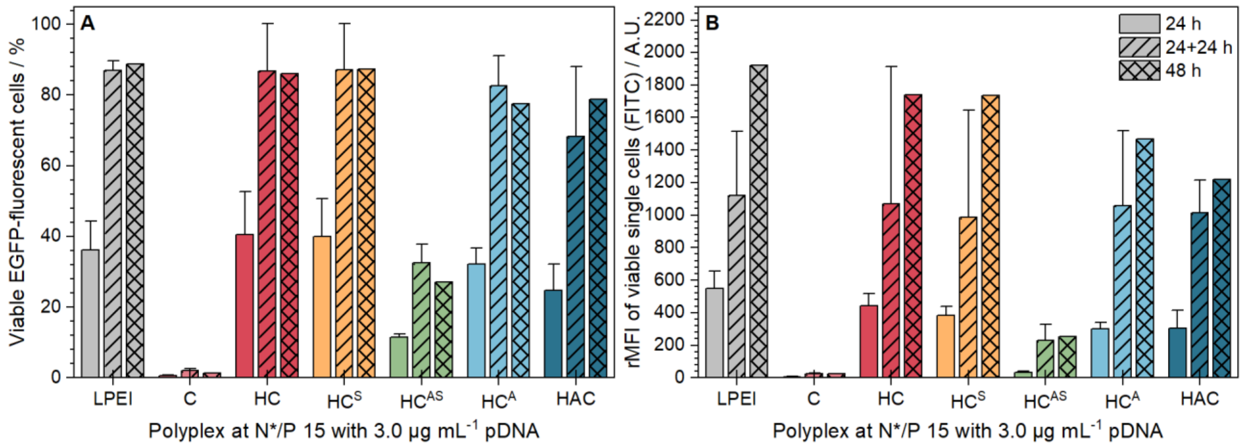

Figure S16. Transfection efficiency with increased pDNA concentration in HEK293T cells.

EGFP expression of viable cells was analyzed *via* flow cytometry following incubation of cells with (layered) polyplexes of mEGFP-N1 pDNA (3.0 µg mL^{-1}) and polymers at N*/P 15 in growth medium for 24 h, for 24 h followed by splitting of cells and medium and further incubation for 24 h, or for 48 h. Values represent mean ± SD of (A) viable, single EGFP positive cells (B) rMFI of all viable single cells (n = 1-3).

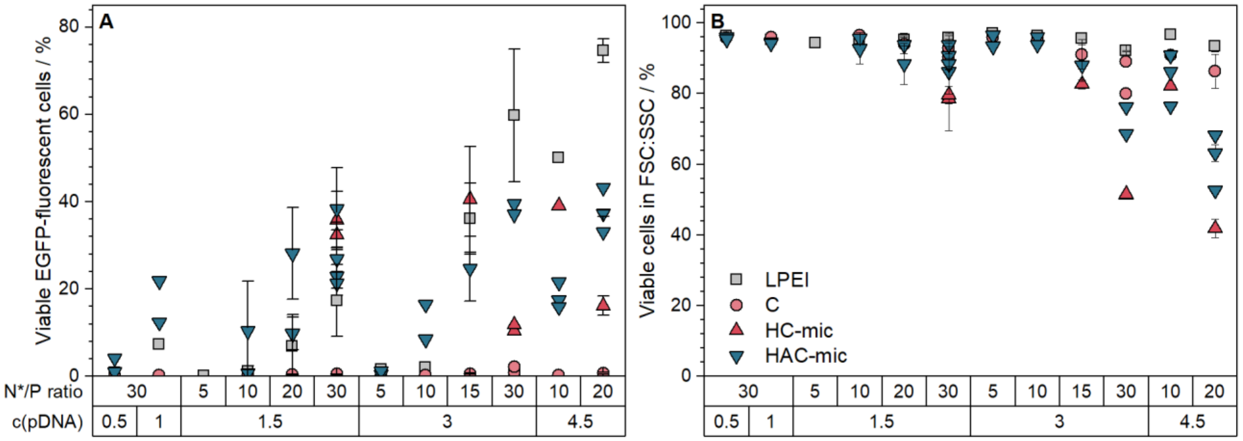

Figure S17. Influence of concentration on transfection efficiency.

Concentration dependent transfection efficiency in HEK293T cells following incubation with polyplexes of mEGFP-N1 pDNA and polymers in D10H for 24 h. Values represent mean ± SD of (A) viable EGFP fluorescent cells or (B) viable cells in FSC/SSC scatter plot (n ≥ 1). Dots of the same color represent different batches of the respective polymer. c(pDNA) in µg mL^{-1}.

Table S8. MFI values of different controls in flow cytometry.

Assay	Conditions	MFI of all viable single cells		
		Only buffer	Only mEGFP pDNA/ YOYO-1 labeled pDNA	pKMyc pDNA + polymers[a]
Transfection efficiency	1.5 µg mL^{-1} pDNA, N*/P 30, 24 h	1693 ± 209	1719 ± 82	2164 ± 483
	1.5 µg mL^{-1} pDNA, N*/P 30, 24+24 h	1753 ± 106	1634 ± 29	2314 ± 517
	1.5 µg mL^{-1} pDNA, N*/P 30, 48 h	1623 ± 200	1415 ± 66	1991 ± 468
Polyplex uptake	1.5 µg mL^{-1} pDNA, N*/P 30, 1 h		2276 ± 280	
	1.5 µg mL^{-1} pDNA, N*/P 30, 4 h		2741 ± 199	
	1.5 µg mL^{-1} pDNA, N*/P 30, 24 h		2433 ± 383	
	1.5 µg mL^{-1} pDNA, N*/P 30, 24+24 h		2461 ± 78	

[a] Mean of all pKMyc pDNA-polymer polyplexes.

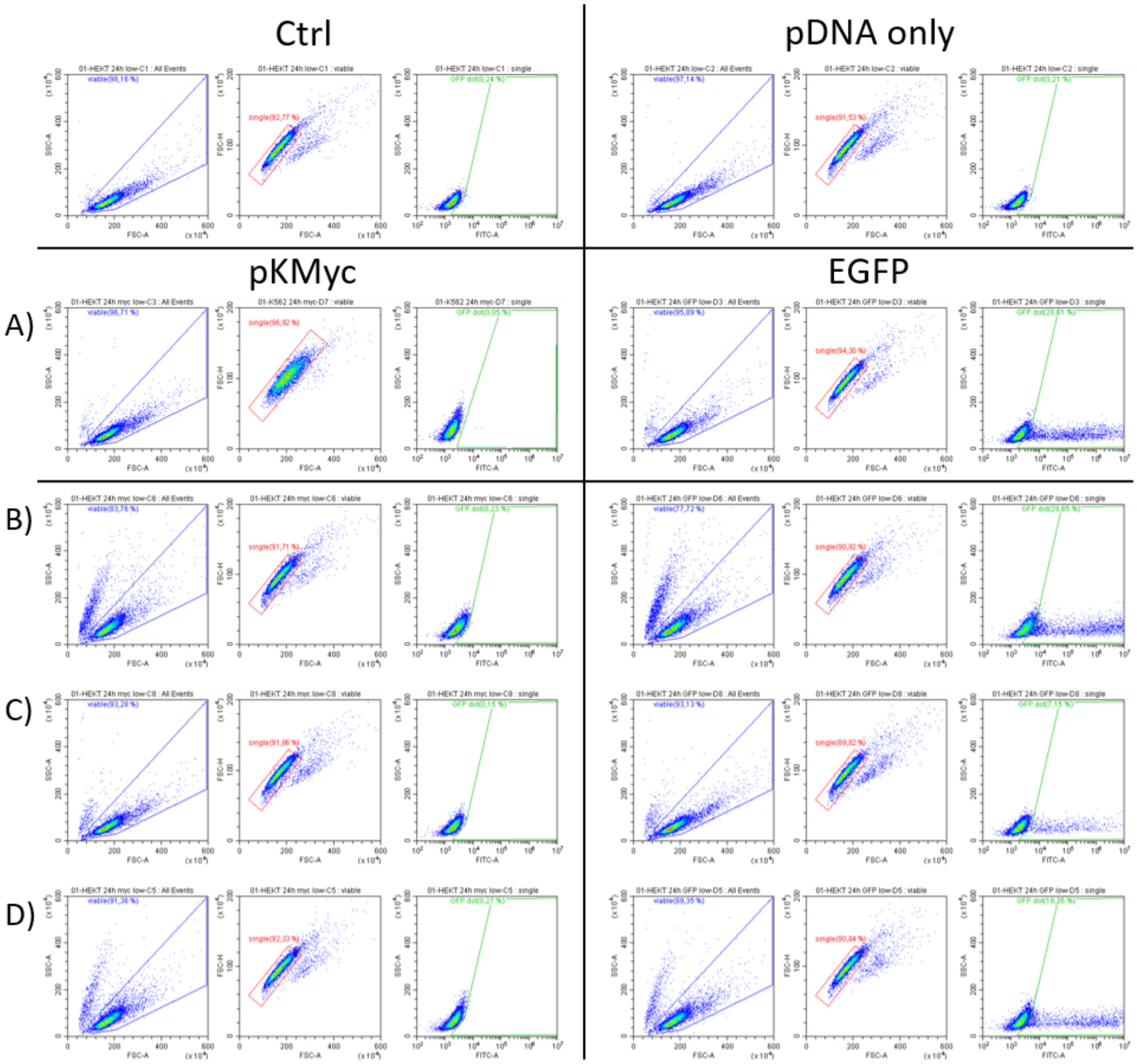

Figure S18. Gating strategy for pDNA transfection using examples of 24 h incubation.
Viable single cells were gated in FSC/SSC and FSC-A/FSC-H dot plots (blue and red gates). Subsequently cells with EGFP fluorescence were discriminated by gating to the respective pKMyc control (green gates). Plots of HEK293T cells incubated with (layered) polyplexes of 1.5 µg mL^{-1} pDNA and (A) LPEI, (B) HC-mic, (C) HC^{AS}, (D) HAC-mic at N*/P 30 are shown.

Polyplex Uptake.

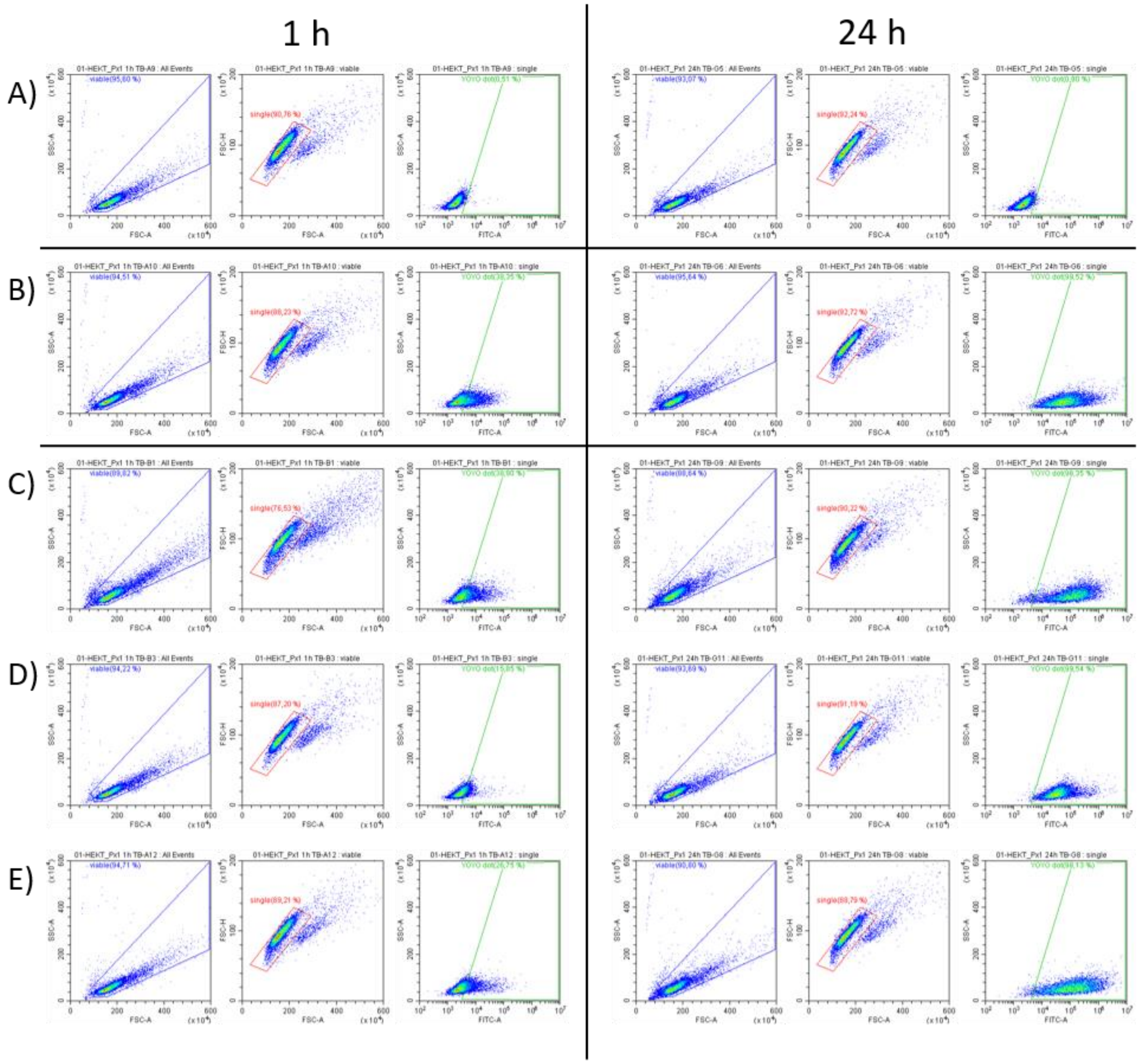

Figure S19. Gating strategy for polyplex uptake using examples of 1 and 24 h incubation. Viable single cells were gated in FSC/SSC and FSC-A/FSC-H dot plots (blue and red gates). Subsequently cells with YOYO-1 fluorescence were discriminated by gating to the pDNA-YOYO-1 control of the respective incubation time (A, green gates). Plots of HEK293T cells incubated with (layered) polyplexes of 1.5 µg mL^{-1} YOYO-1 labeled pDNA and (B) LPEI, (C) HC-mic, (D) HC^{AS}, (E) HAC-mic at N*/P 30 are shown.

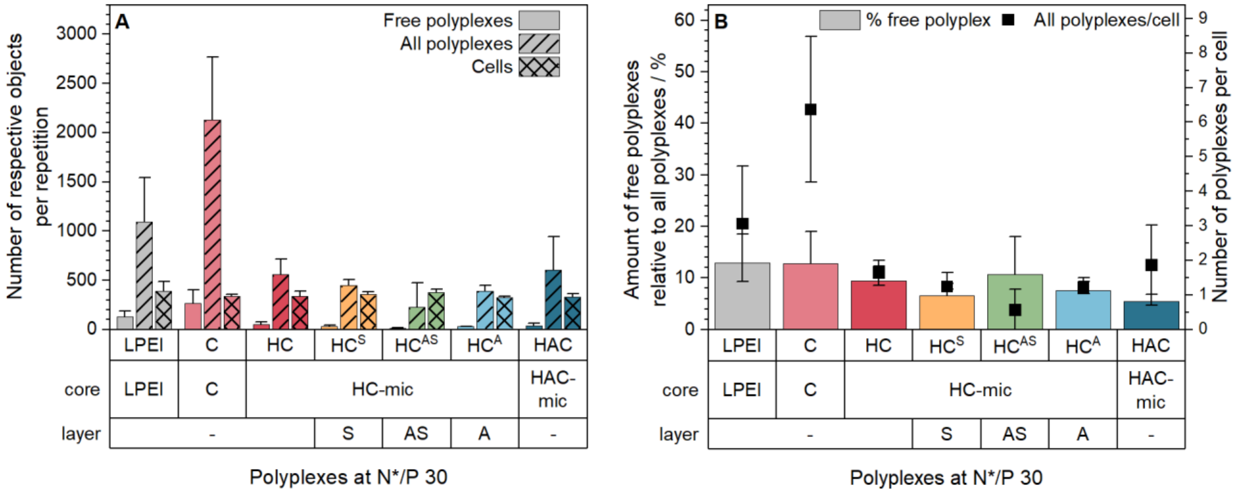

Figure S20. Quantitative analysis of polyplex uptake by CLSM.

HEK293T cells were stained for plasma membrane and membrane-associated organelles with CMDR-PM followed by incubation with (layered) polyplexes of YOYO-1-labeled pDNA and polymers at N*/P 30 in D10H for 1 h. Live cell imaging was performed following further staining of the cells with Hoechst 33342 for the nuclei and again with CMDR-PM. YOYO-1 fluorescence outside the cells was quenched by addition of trypan blue to a final concentration of 0.04% just before imaging. Values were obtained by image analysis of all acquired images (Figure S21) using the analysis wizard of ZEN 3.1 (Zeiss, Germany) and represent mean ± SD (n = 3) of (A) the raw counts of the respective segmented features, and (B) the respective relative calculations (see additional methods section).

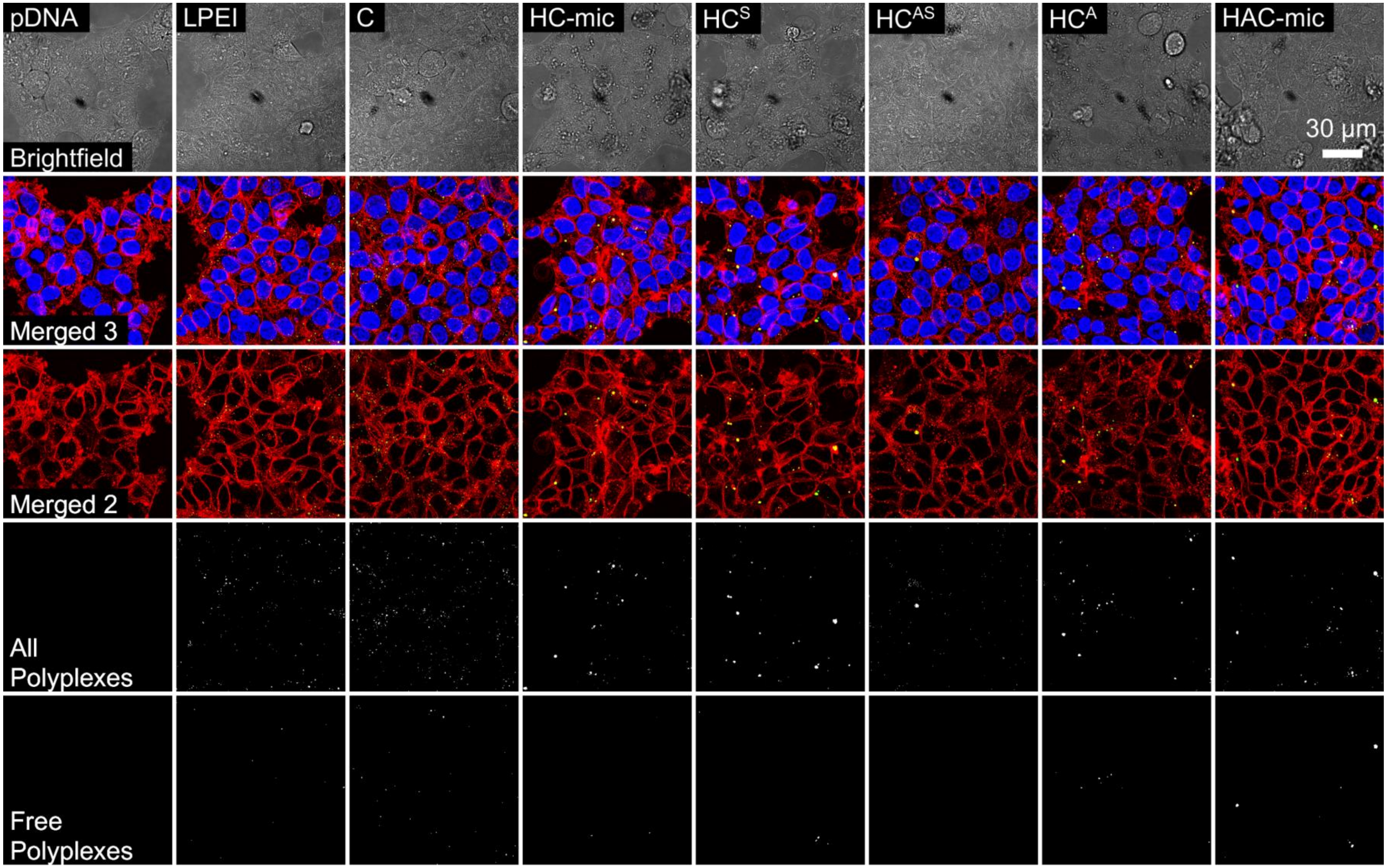

Figure S21. CLSM images of polyplex uptake by HEK293T cells.

Representative images of HEK293T cells stained for plasma membrane and membrane-associated organelles (red) with CMDR-PM followed by incubation with (layered) polyplexes (green) of YOYO-1-labeled pDNA and polymers at N*/P 30 in D10H for 1 h. Live cell imaging was performed following further staining of the cells with Hoechst 33342 for the nuclei (blue) and again with CMDR-PM. Yellow dots indicate colocalization of CMDR-PM and YOYO-1. The images of the free polyplexes were generated by subtraction of the respective binary colocalization image from the binary polyplex image obtained during image analysis.

Calcein Release.

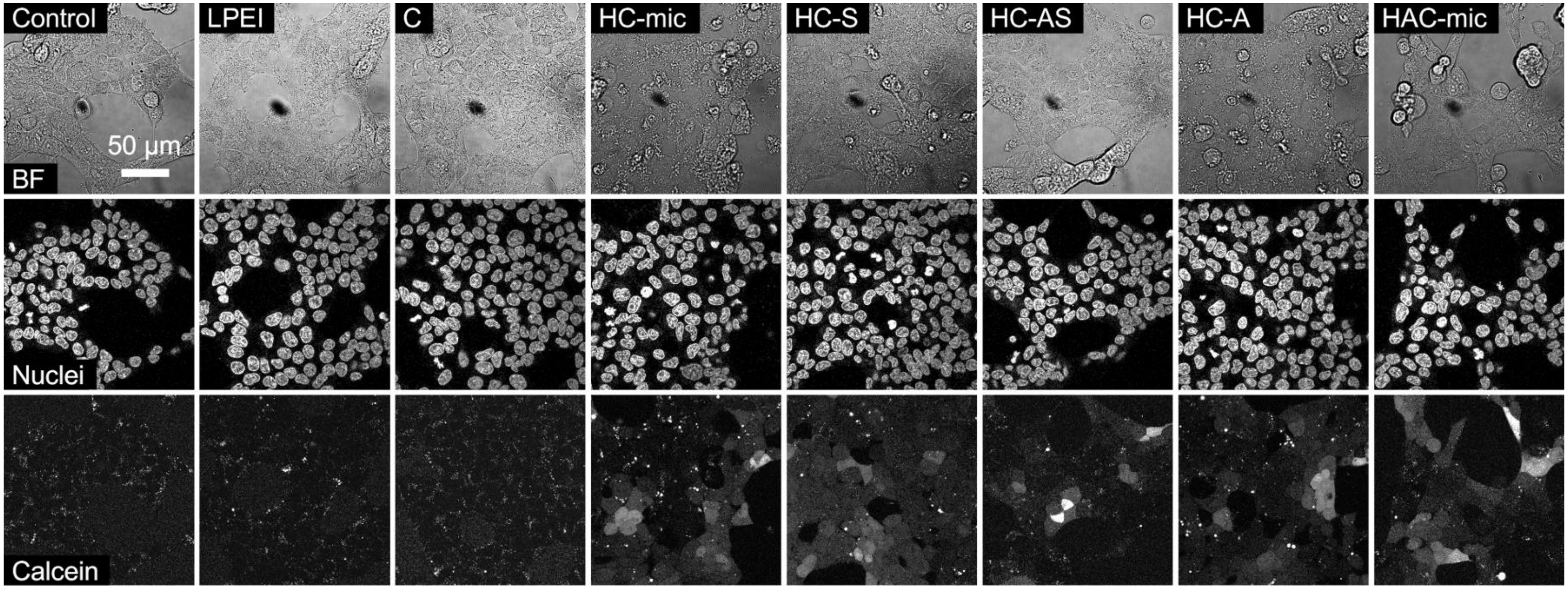

Figure S22. Brightfield (BF) and gray images of HEK293T cells for calcein CLSM study.

HEK293T cells were incubated with (layered) polyplexes of pDNA and polymers at N*/P 30 in D10H for 1 h. Just before the addition of polyplexes, calcein was added to a final concentration of 25 µg mL^{-1}. Cells were washed twice with warm FC-buffer before the

addition of fresh warm D20 and staining of nuclei with Hoechst 33342. Images of the T-PMT channel are shown. Colored images of the calcein and the Hoechst 33342 channels can be found in the main article.

REFERENCES

1. Larnaudie SC, Brendel JC, Jolliffe KA, Perrier S: **Cyclic peptide–polymer conjugates: Grafting-to vs grafting-from.** *J Polym Sci, Part A: Polym Chem* 2016, **54:**1003-1011.
2. Richter F, Martin L, Leer K, Moek E, Hausig F, Brendel JC, Traeger A: **Tuning of endosomal escape and gene expression by functional groups, molecular weight and transfection medium: a structure-activity relationship study.** *J Mater Chem B* 2020, **8:**5026-5041.
3. Richter F, Mapfumo P, Martin L, Solomun JI, Hausig F, Frietsch JJ, Ernst T, Hoeppener S, Brendel JC, Traeger A: **Improved gene delivery to K-562 leukemia cells by lipoic acid modified block copolymer micelles.** *Journal of Nanobiotechnology* 2021, **19:**70.
4. Schindelin J, Arganda-Carreras I, Frise E, Kaynig V, Longair M, Pietzsch T, Preibisch S, Rueden C, Saalfeld S, Schmid B, et al: **Fiji: an open-source platform for biological-image analysis.** *Nat Methods* 2012, **9:**676.
5. Catrouillet S, Brendel JC, Larnaudie S, Barlow T, Jolliffe KA, Perrier S: **Tunable Length of Cyclic Peptide–Polymer Conjugate Self-Assemblies in Water.** *ACS Macro Lett* 2016, **5:**1119-1123.
6. Bauer M, Lautenschlaeger C, Kempe K, Tauhardt L, Schubert US, Fischer D: **Poly(2-ethyl-2-oxazoline) as Alternative for the Stealth Polymer Poly(ethylene glycol): Comparison of in vitro Cytotoxicity and Hemocompatibility.** *Macromol Biosci* 2012, **12:**986-998.
